# Intermittent control and retinal optic flow when maintaining a curvilinear path

**DOI:** 10.1038/s41598-025-02402-3

**Published:** 2025-05-29

**Authors:** Björnborg Nguyen, Ola Benderius

**Affiliations:** Department of Mechanics and Maritime Sciences, Chalmers University of Technology, 412 96 Göteborg, Sweden

**Keywords:** Retinal optic flow, Visual motion flow, Heading perception, Intermittent control, Ballistic correction, Human perception, Human control, Human sensorimotor model, Sustained sensorimotor model, Perception, Cognitive control, Motor control, Navigation

## Abstract

The topic of how humans navigate using vision has been studied for decades. Research has identified the emergent patterns of retinal optic flow from gaze behavior may play an essential role in human curvilinear locomotion. However, the link towards control has been poorly understood. Lately, it has been shown that human locomotor behavior is corrective, formed from intermittent decisions and responses. A simulated virtual reality experiment was conducted where fourteen participants drove through a texture-rich simplistic road environment with left and right curve bends. The goal was to investigate how human intermittent lateral control can be associated with the retinal optic flow-based cues and vehicular heading as sources of information. This work reconstructs dense retinal optic flow using a numerical estimation of optic flow with measured gaze behavior. By combining retinal optic flow with the drivable lane surface, a cross-correlational relation to intermittent steering behavior could be observed. In addition, a novel method of identifying constituent ballistic correction using particle swarm optimization was demonstrated to analyze the incremental correction-based behavior. Through time delay analysis, our results show a human response time of approximately 0.14 s for retinal optic flow-based cues and 0.44 s for heading-based cues, measured from stimulus onset to steering correction onset. These response times were further delayed by 0.17 s when the vehicle-fixed steering wheel was visibly removed. In contrast to classical continuous control strategies, our findings support and argue for the intermittency property in human neuromuscular control of muscle synergies, through the principle of satisficing behavior: to only actuate when there is a perceived need for it. This is aligned with the human sustained sensorimotor model, which uses readily available information and internal models to produce informed responses through evidence accumulation to initiate appropriate ballistic correction, even amidst another correction.

## Introduction

Vision is arguably one of the most complex perception senses enabling animals to navigate and exploit the ever-dynamic surrounding world as we understand it^1–3^. Although there is a scientific consensus that vision perception plays a critical role, it is not yet fully understood how humans use it to guide, propel, or control their egomotion on foot^4,5^ or in vehicles. It has been proposed that visual flow, more specifically the *retinal optic flow*, is exploited guiding the planar curvilinear locomotion in a textured environment^6–13^, e.g. riding a bicycle or driving a road vehicle. It has often been argued that task-driven gaze fixation transforms and stabilizes retinal optic flow patterns to counteract the chaotic relative motion that may occur during locomotion^11,14^ and further due to anticipation of trajectory planning^4,15–17^. Our work integrates various key findings across scientific domains and demonstrates how visual stimuli, such as retinal optic flow, may result in an intermittent ballistic correction in human locomotor control. This work offers insight into how visual input may relate to action output response for correcting egomotion.

It is an ongoing discussion whether human locomotor behavior can be solely described through a *strong online* approach, a *strong model-based* approach, or as a hybrid composite of both^18^. A strong online control mechanism is characterized by solely relying on the immediate available external information to act upon. In contrast, a strong model-based approach uses an internalized model to process the external information, transform it, update one’s belief, or predict the internal states, which are then used to act upon. Recently, there has been increasing evidence that humans must have internalized models to explain observed predictive behavior in humans^17,19,20^ (model-based control). Simultaneously, other research implies that initial muscle motor control processes are completed during the motor planning phase in the human brain^21–25^, suggesting that humans complete the initial action response *before* any other auxiliary higher cognitive processes as prediction and planning are fully completed (online control).

*Intermittent control* as a phenomenon is commonly found in psychology and biomechanical contexts. This biomechanical control emerges from the fact that muscles are only capable of generating force through contraction, often around a joint. Thus biological systems are evolutionarily optimized for energy efficiency, co-contraction, muscle synergy, etc. Since energy is a valuable resource, things ought to change *only* when there is a sufficient need for it. Despite intermittent control being an established topic in experimental psychology^26,27^, it is only recently gaining attraction outside of its field when studying human control behavior^28,29^. In contrast to optimal control and classical control theory^30^, human control behavior should be understood as an intermittent control system where corrections are intermittently applied via *trains of ballistic movements* to achieve a task^21,27^. This has been successfully developed, applied, and demonstrated through computational model frameworks^28,29,31^. It has been found that the human-operated steering wheel for lateral control in vehicles also exhibits the same intermittency property^28^.

In neuromuscular modeling using the *minimum-jerk model*^32^, these ballistic correction movements correspond to so-called *reaching* movements, rapid aimed limb movement from point to point, with characteristics of sigmoid positional and bell-shaped velocity profiles^33,34^. To achieve one single ballistic reaching movement, a set of building blocks of neural and muscle networks, or *muscle synergies*, are activated and recruited^35–38^. It is believed that the central nervous system uses these muscle synergies to simplify the coordination and control of complex movements to alleviate the cognitive load. This is done by mapping initial states and task-specific goals to time-varying activation of muscle synergies on a higher level, contrary to individually micro-controlling each muscle primitive in a centralized manner^35,36^.

Various research disciplines have contributed significantly to our understanding of human locomotor control behavior. However, these contributions are often made in isolation in their respective research domain without any further integration into other fields. There have been limited attempts to incorporate these individual findings into a more unified and harmonized understanding of human locomotor control. Unification of these ideas paints the larger picture and extrapolates their findings outside of their place of inception and driving science forward^39,40^. In this work, we investigate and report the findings observed in human driving behavior in curvilinear paths in a visually textured environment, bridging the gaps across the research fields. In particular, our objective is to study in detail how retinal optic flow can serve as a perception cue for intermittent control, e.g. how the perception of visual motion may trigger a ballistic locomotor correction response. Emerging from this, the following research questions are posed:

1. How well can intermittent control describe human steering behavior by quantifying overlapping ballistic steering corrections?
2. What are the human response times, from visual stimuli (retinal optic flow and heading) onset to steering correction onset?
3. How does the human locomotor behavior change when removing vehicle-fixed objects visually?
4. How can visual perception cues trigger or correlate with ballistic correction as reaching?

In order to investigate the posed research questions, our work identifies and deals with three significant hurdles of quantification: retinal optic flow, ballistic adjustments in human intermittent control, and human mental processing latency. In the first part, the retinal optic flow field as perceived by the locomoting agent can be qualitatively reconstructed using a set of idealized assumptions of smooth pursuit and experimental data. In the second part, ballistic adjustment is derived based on the theories of intermittent control and demonstrated on the one-dimensional steering wheel angle in our work. In addition, our work presents a novel experimental method using a stochastic approach to identify each ballistic correction in experimental data with complex overlapping ballistic corrections. Finally, these results allow for an estimation of human cognitive latency using cross-correlation analysis of perceived stimuli to a correctional action for lateral control. In addition, locomotor performance is compared by offering a fully unobstructed view of the scene by making the steering wheel invisible. Doing so may provide insight into how the perception of the ego-state impacts human locomotor control performance.

## Background

### Human perception in curvilinear path

For decades, researchers have long debated the intricate details of what and how humans perceive the environment for navigation^9,41,42^. One such debate is whether retinal optic flow, without recovering the heading or exploiting extra-retinal information, may be used in curvilinear locomotor control^6,7,12,42,43^. Although it has been shown mathematically^44^ that it is possible to make the correct steering judgments by relying solely on the immediate available retinal optic flow, it has yet to be decisively concluded to be the case. It is still poorly understood if these steering judgments are informed solely on retinal optic flow, combined with extra-retinal information, or allocentric cues alone. One contributing factor to this problem is the lack of relevant high-quality data to complete the debate. This has effectively resulted in the status quo in research regarding human sensorimotor models incorporating gazing until recently with the rapid development of modern eye-tracking systems.

**Fig.1:**
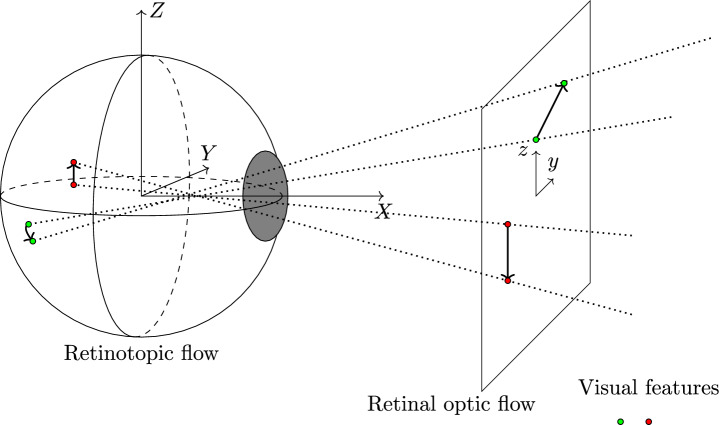
Retinal optic flow and retinotopic flow. The illustration demonstrates how visual motion flow is projected to the retina in the eye, creating the retinal optic flow or retinotopic flow sensation the observer experiences. The retinal optic flow field accounts for the rotational eye movements exhibited during gaze fixation and smooth pursuit, achieving stabilization of the retinal image. The main difference between retinal optic flow and retinotopic flow is the stereographic projection, i.e. projection to the sphere skewing coordinate system.

Meanwhile, visual flow has been widely adopted across scientific and engineering domains. This has created ambiguity as researchers across domains have used the terms *optic flow* and *retinal optic flow* (or retinal flow in early literature) interchangeably. In the literature, optic flow is often considered in terms of the locomotor axis or the velocity vector of the observer. In this work, the optic flow will be computed and referred to the visual flow fields fixed with respect to the head-fixed coordinate system (due to the virtual reality–head-mounted display). The retinal optic flow^10,11^ here will refer to the emergent patterns of visual motions that would be projected to the human eye but omitting the *stereographic projection* (projection to a sphere). This mainly accounts for expressed eye movements. If the final stereographic projection to the retina is performed, this could be referred to as the *retinotopic flow fields* or visual flow in *retinotopic coordinates* in the neurocognitive science. This can be imagined with a fixed coordinate system to the eye as the visual motion flow is projected onto the retina, see Fig. 1. It is foremost the retinotopic flow field stimulus that the observer experiences. However, in our work, the retinal optic flow is mainly considered without loss of generality when the stereographic projection is omitted.

Researchers advocating for retinal optic flow as the main perceptual cue for curvilinear locomotor control have proposed a simplified perception-control heuristic^6,7,9^. The proposal goes in steps: (a) Fixate the gaze close to the intended path, (b) the directions of the retinal optic flow over the intended path will then indicate understeering or oversteering, and finally (c) if the retinal optic flow directions of the intended path are pointing downward, then the future path and intended path coincide and no further correction is needed. This behavioral heuristic can simply be formulated as *“you look where you are going”* and *“you go where you are looking”*. Interestingly, the gazing behavior implied by the stated heuristic may be a *conditioned* behavior as taught by advanced driving instructors and possibly exhibited by licensed (experienced) drivers^7^. There is a significant demand for proper steering maintenance during critical moments, such as curve bends, resulting in a larger cognitive load^15^. This may suggest or motivate why such a strategy is deployed to cope with this.

**Fig. 2:**
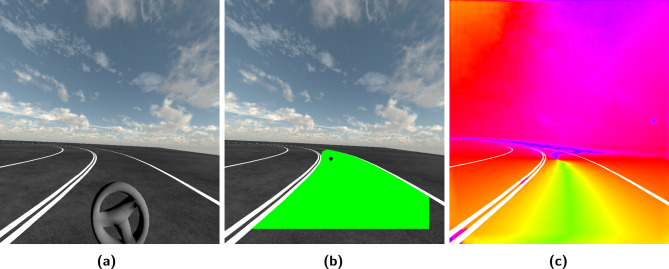
Visual input, image processing, and data visualization. Panel **(a)** Demonstrates the point of view of a research participant in virtual reality. The virtual world is made visually simple. It consists of a textured sky, textured ground with painted lane markings, and a steering wheel. This particular time instance is captured during the left curve bend negotiation. Panel **(b)** shows the post-processed data rendered on top of the raw image view without the steering wheel. In the image, the gaze point of the participant is illustrated with a black crosshair, and the detected lane segment is colored as a green area. Panel **(c)** shows retinal optic flow stimulus is reconstructed by combining motion analysis of temporal consecutive images and gaze data. The colors represent the direction of the optic flow vectors in the field, see Supplementary Fig. S1 for further detailed explanation and illustration. The green color is equivalent to the retinal optic flow pointing in the downward direction. For visual reference, the lane markings are painted white on top of the flow for this illustration. Videos of the author driving are linked here: https://youtu.be/rW_X9OZ3mRM and https://youtu.be/d0xtk2d4wFk (with GUI).

From a research point of view, the challenge lies in quantifying the retinal optic flow of the *intended path* of the agent as stated by the simplified heuristic if such a quantity exists. Given the task that the agent is supposed to maintain their current future path so that this path lies within their designated road lane, then the designated road lane should contain their internalized projected intended path. This still holds when the agent cuts corners when locomoting in a curve bend. Then the intended path is shifted closer to the corner side of the lane but still within the boundaries of the designated road lane. For this work, it will be assumed that the designated road lane visually contains the intended path of the agent as the task is to stay in the lane while completing the task. To then construct a quantity as described by the heuristic, the *spatial circular mean angle of retinal optic flow over the intended path* becomes

1 $$\bar{\theta }_{{{\text{rof}}}} = \arg \left( {\sum\limits_{{\theta _{{{\text{rof}}}} \in \Omega _{{{\text{lane}}}} }} {\exp } \left( {i\theta _{{{\text{rof}}}} } \right)} \right),$$

where $\theta _\text {rof}$ is the directional angle of the dense retinal optic flow field (see the visualization of retinal optic flow in Fig. 2c and further Fig. S1 in Supplementary Materials), $\Omega _\text {lane}$ is the set containing the dense visual flow of the visual lane (corresponds to the green area in Fig. 2b), and *i* is the imaginary unit. In this work, this quantity will be referred to as *retinal optic flow angle* instead of the spatial circular mean angle of the retinal optic flow over the intended path. Note that the naive arithmetic mean is not directly applicable to cyclic quantities, such as angles, due to the cyclic boundaries. The retinal optic flow angle can thus be understood as some extended computational quantity of the perceived *retinal flow patterns* of the intended path of the agent as described in the literature^6,7^. Here, the retinal optic flow angle is exclusively based on visual information, such as the retinal optic flow and lane perception without explicitly utilizing external information such as vehicular heading or vehicular lateral lane position.

For completeness, the locomotor heading cue will be expressed as the angle between the road curvature tangent to the vehicular heading as

2 $$\begin{aligned} \theta _\text{ h } = \theta _\text {road} - \theta _\text {veh}, \end{aligned}$$

and vehicular lateral displacement as the lateral offset (lateral relative to the road curvature) from the middle line of the designated lane as

3 $$\begin{aligned} y_\text{ l } = y_\text {lane} - y_\text {veh}. \end{aligned}$$

The heading deviation $\theta _\text{ h }$ is closely related to lateral displacement velocity $\dot{y}_\text {l}$. Disregarding the advanced vehicle and tire dynamics, the lateral displacement velocity may be simplified as

4 $$\begin{aligned} \dot{y}_\text {l} = ||\varvec{v}_\text {veh}||_2 \sin {\theta _\text{ h }} \approx ||\varvec{v}_\text {veh}||_2 \theta _\text{ h } \end{aligned}$$

where the last approximation is valid for small angles of $\theta _\text{ h }$.

### Human motor control and intermittent control

To better understand *if* and *when* stimuli that trigger an expressed movement as a response, one needs to explore and understand the kinematics and characteristics of the expressed limb movements. When humans exert a voluntary rapid aimed limb movement in space, the movement is characterized using the minimum-jerk model^32^ of having a sigmoid positional curve as a distance to a target point^45^ and a bell-shaped velocity^33^ profile. This is also known as *reaching* movement in the fields of neuroscience^34,46,47^. In addition, observations made in experimental movement data reveal that velocity profiles of reaching often have a heavy tail^34^, originating from slower homing movement near the target point. However, for this work, the bell-shaped velocity profiles will be considered symmetric; see the Supplementary Material for further analysis and motivation. If the reaching can be considered symmetric, then one simple or ballistic reaching movement (ballistic correction) can be approximated as a symmetric Gaussian function

5 $$\begin{aligned} {\dot{\delta }}_\text{ b }(t,a_\text{ I }, \mu _\text{ I }, \sigma _\text{ I}) \triangleq a_\text{ I } \exp { \frac{-(t-\mu _\text{ I})^2}{2\sigma _\text{ I}^2}}, \end{aligned}$$

where $a_\text{ I }$ is the correction strength, $\mu _\text{ I }$ the mode of correction, and $\sigma _\text{ I }$ the correction rate parameter. The correction strength parameter $a_\text{ I }$ may be physically interpreted as how strong the applied correction is and its direction, similar to an amplitude coefficient (indicated direction and length of the correction mode in Fig. S3 in Supplementary Materials). The correction mode parameter $\mu _\text{ I }$ determines the timeliness of correction, shifting the correction forward or backward in time (placement in time of the correction modes in Fig. S3 in Supplementary Materials). Lastly, the correction rate parameter $\sigma _\text{ I }$ dictates how rapidly the correction is applied, smaller values shorten the duration and larger values prolong the duration.

In reality, however, the reaching movements are rarely cleanly executed by one correction, but by a highly complex train of corrections where they may overlap each other. The resulting complex movement of individual corrections can be described by the principle of superposition

6 $$\begin{aligned} {\dot{\delta }} (t) = \sum _{(a_\text{ I }, \mu _\text{ I }, \sigma _\text{ I}) \in \Omega _{{\dot{\delta }}}} \dot{\delta }_\text{ b }(t,a_\text{ I }, \mu _\text{ I }, \sigma _\text{ I}) \end{aligned}$$

where $\Omega _{{\dot{\delta }}}$ is a $3\times N_\delta$ matrix

7 $$\begin{aligned} \Omega _{{\dot{\delta }}} =\begin{pmatrix} a_0 & a_1 & \dots & a_{N_\delta -1} \\ \mu _0 & \mu _1 & \dots & \mu _{N_\delta -1} \\ \sigma _0 & \sigma _1 & \dots & \sigma _{N_\delta -1} \\ \end{pmatrix}. \end{aligned}$$

Each column in $\Omega _{{\dot{\delta }}}$ contains the parameters $(a_\text{ I }, \mu _\text{ I }, \sigma _\text{ I})$ for a single ballistic correction. Refer to Supplementary Fig. S2 for illustrative examples of simple and overlapping complex reaching movement patterns.

Followed by the properties of the Gaussian function, the onset and offset of each intermittent correction movement can then be clearly defined as

8 $$\begin{aligned} t_\text {I,\{on,off\}} \triangleq \mu _\text{ I } \mp 2\sigma _\text{ I } \end{aligned}$$

by the given correction mode $\mu _\text{ I }$ and correction rate parameter $\sigma _\text{ I }$. Following this convention, the total duration of the movement is defined as $4\sigma _\text{ I }$ which encompasses approximately 95.4 % of the complete theoretical movement.

The positional distance function is simply the primitive function of the velocity profile of the correction as defined in Eq. (5)

9 $$\begin{aligned} \delta _\text{ b }(t,a_\text{ I }, \mu _\text{ I }, \sigma _\text{ I}) = \int a_\text{ I } \exp {\frac{-(t-\mu _\text{ I})^2}{2\sigma _\text{ I}^2}} \text{ d }t = a_\text{ I } \sigma _\text{ I }\sqrt{\pi /2} ~\text {erf}(\frac{t-\mu _\text{ I }}{\sqrt{2}\sigma _\text{ I }})+C \end{aligned}$$

where $\text {erf(\dots )}$ is the Gauss error function and can not be expressed by elementary functions, and *C* is some constant determined by a known condition in position (usually a boundary condition). The full travel distance of the ballistic movement correction is then

10 $$\begin{aligned} \Delta \delta _\text{ b } (a_\text{ I }, \sigma _\text{ I}) = \int _{-\infty }^{\infty } \dot{\delta }_\text{ b }(t,a_\text{ I }, \mu _\text{ I }, \sigma _\text{ I}) \text{ d } t = a_\text{ I } \sigma _\text{ I } \sqrt{2\pi }. \end{aligned}$$

The travel distance is fully determined by the correction strength and the speed parameter, which is to be expected. Examples of theoretical ballistic corrections for both cases of single correction and complex overlapping corrections are shown in Fig. S2 with the defined onset of offset timing.

A human-operated steering wheel shares the same fundamental characteristic as human reaching movements of a sigmoid positional curve and the bell-shaped velocity profile^28,29^. Under the assumption of normal steering handling and sufficiently small steering wheel angles to avoid hand shuffling, the ballistic correction duration parameter for the steering wheel was empirically found to be $\sigma _{{{\text{ I }}}} \in [0.0707{\kern 1pt} {\text{s}},0.1732{\kern 1pt} {\text{s}}]$, similar to other human driver modeling framework^29^. By these constraints, one single ballistic steering correction movement duration is then within 0.28 s to 0.69 s.

### Human cognitive response time

**Fig. 3:**
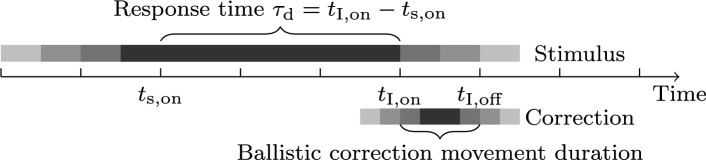
Intermittent control timeline for a single correction. The figure illustrates the timeline of an intermittent control where a ballistic correction is triggered by a stimulus at the stimulus onset time $t_\text {s,on}$. The perceived need for an action is highlighted with gradients of red. There is also a small time window to sample the perceived error, a process known as evidence accumulation. Once this perceived error becomes large enough, a ballistic correction is triggered as a response at the time $t_\text {I,on}$. If the expressed response is correct and proper, it reduces the need for action.

In between when humans receive a stimulus to the moment of exerting a response movement, are the neurocognitive processes and mechanisms that form the pathways in the central nervous system. It may generally be understood as information propagating through (a) the perception pathway to make sense of the stimulus, then (b) the control pathway to form, plan, and predict movements, and finally, (c) the muscle pathway to execute the movements. Assuming the signal propagates through these pathways, then the total response time is defined by the sum of the cognitive processing time^29^ as

11 $$\begin{aligned} \tau _{\text{ d }} \triangleq \tau _{\text{ p }} + \tau _{\text{ c }} + \tau _{\text{ m }} = t_\text {I,on} - t_\text {s,on}, \end{aligned}$$

where $\tau _\text{ p }$ is the cognitive perception processing time delay, $\tau _\text{ c }$ the control decision time delay, and $\tau _\text{ m }$ the muscular activation time delay for muscle recruitment (movement onset. This should match the time difference from stimuli onset $t_\text {s,on}$ to movement onset $t_\text {I,on}$, see Fig. 3 for a timeline illustration for a single correction. Control decision time delay $\tau _\text{ c }$ is considered general and may encompass the processes using internalized modeling such as higher cognitive functions, e.g. predictive behavior or movement planning, and may span beyond seconds before a response is initiated. Methodically characterizing this control factor is challenging due to its general nature and is heavily dependent on the internalized model and the higher-level intentions of the agent. For the purpose of this work, only immediate stimulus-triggered responses will be considered, which effectively renders $\tau _{{{\text{ c }}}} \approx 0{\kern 1pt} {\text{s}}$. In neuroscience, previous experimental results based on both humans and primates have suggested numerical estimations of delay timings to be $\tau _{{{\text{ p }}}} \in [0.074{\kern 1pt} {\text{s}},0.142{\kern 1pt} {\text{s}}]$ for visual perception^29,48,49^ and $\tau _{{{\text{ m }}}} \in [0.050{\kern 1pt} {\text{s}},\,0.122{\kern 1pt} {\text{s}}]$ for motor cognition^29,50,51^.

To experimentally determine the response time (time delay) of a system, one can analyze the cross-correlation which is a common technique in signal processing in the engineering domain. To identify the system response time $\tau _\text{ d }$, one finds the time delay $\tau$ that maximizes cross-correlation between input and output signals of the system as

12 $$\begin{aligned} \tau _\text{ d } = \mathop {\mathrm {arg\,max}}\limits _{\tau } (C\star S)(\tau ) \end{aligned}$$

where $(X\star Y)(\tau )$ is the cross-correlation (similar to convolution but without time reversal) between the control output signal *C* and stimuli input signal *S* for a time delay $\tau$. Here a zero-normalized cross-correlation will be used

13 $$\begin{aligned} (C\star S)(\tau ) = \rho _\text {p}(\tau ) = \frac{\text{ E }[(C(t)-\text{ E }[C(t)])(S(t-\tau )-\text{ E }[S(t-\tau )])]}{\sigma _{C(t)} \sigma _{S(t-\tau )}} \end{aligned}$$

where $\text{ E }[X]$ is the expected value of *X* and $\sigma _X$ is the standard deviation of *X*. This brings the two compared signals to zero-mean and normalizes the correlation to the interval $\rho _\text{ p } \in [-1,1]$. This is also known as the *Pearson correlation coefficient* in statistics, which naively measures the linear relationship of the two signals.

Combining this and the intermittency property (see Eq. 8), it is possible to formulate a set of expectations regarding the latency results depending on the human cognitive processing for steering control can be characterized as online-based versus internal model-based. For the former case of online control, a strong peak should be identified in the cross-correlation, as the actions are mainly driven by readily available stimuli. This peak should be located at $\tau _d \approx 0.15$ as suggested by previous results in neurocognitive science. For the latter case of model-based control, it is suggested that the control process integrates previous experience and information to form the actions, i.e. $\tau _c > 0$ of unknown distribution, adding a heavier tail to the cross-correlation after $\tau _d \approx 0.15$.

## Methods

### Ethics

**Fig. 4:**
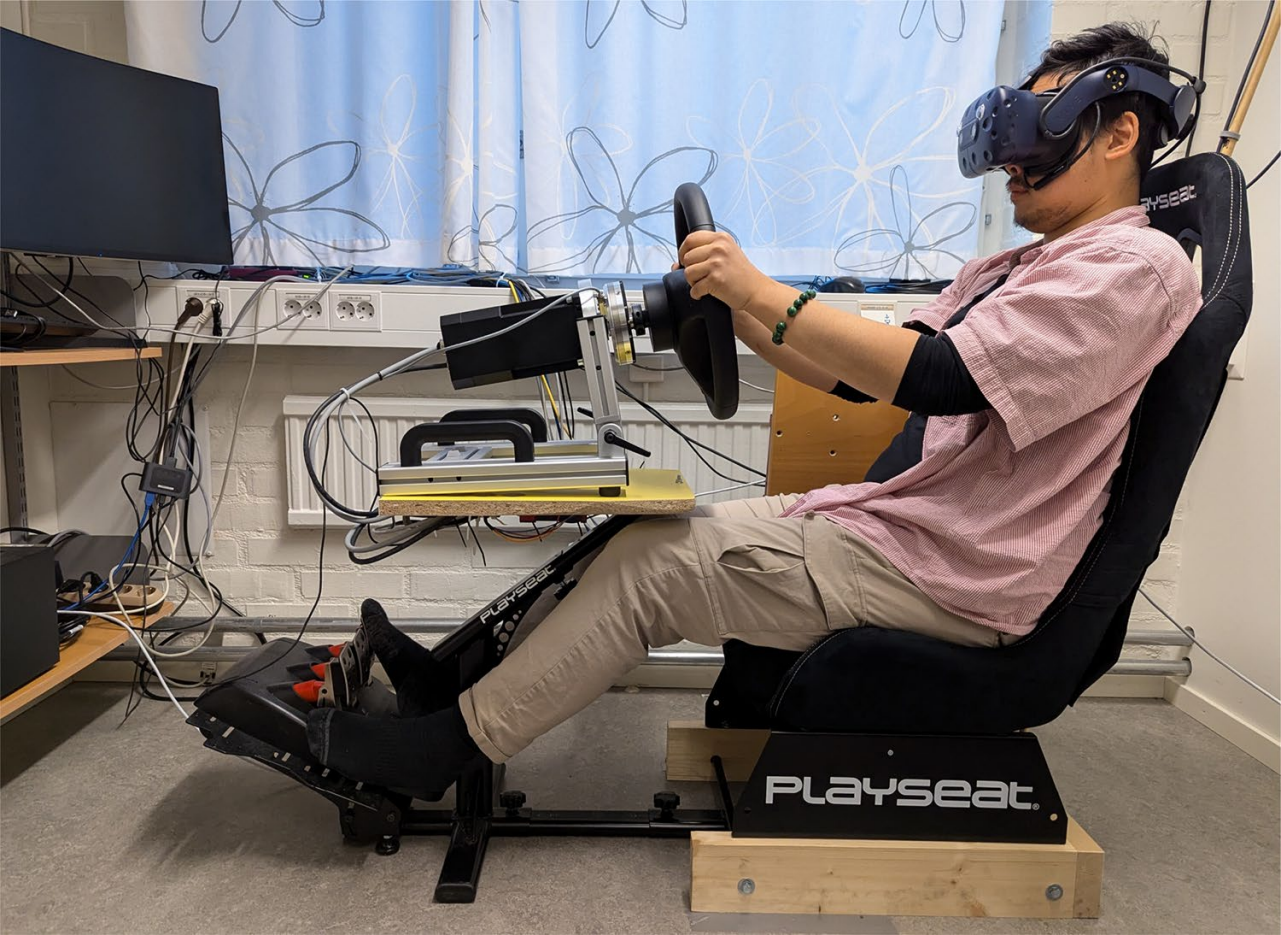
The experimental hardware setup with one of the authors demonstrating as a participant. The image depicts a fully assembled experimental setup consisting of a virtual reality head-mounted display with an eye-tracking system (not visible), a racing-sim chair with a mounted steering wheel rack with a direct drive force-feedback feature, and a pedal set system. There are also VR base stations in the room tracking the head movements of the research participant.

Informed consents were obtained from the research participants prior to the experiment to collect, process, and publish the research findings. The author depicted in Fig. 4 consented to the publication and use of the image. This research project has been reviewed and approved by the *Swedish Ethical Review Authority* (reference number: 2023-03453-01) in accordance with the declaration of Helsinki and the Swedish Ethical Review Act.

### Research participants

A total of fourteen naive healthy adult research participants with normal or corrected-to-normal vision were recruited for the research experiment (six females, eight males, $\mu _\text {age} \approx 25.36\,\hbox {yrs}$, $\sigma _\text {age} \approx 5.29\,\hbox {yrs}$, ranging 18–38 yrs). Three research participants were part of the pilot phase of the study and were excluded from this work.

No formal requirements or qualifications of a driving license, past driving experience, or previous video gaming experience were imposed during recruitment. From this, a total of 110 experimental trials were procured. The research participants were also asked to provide additional information about their driving, video gaming habits, and basic demographic data through a digital survey in connection to the experiment. All participants reported to be in good health at the time of the experiment. There were some reported cases where research participants became slightly motion sick due to exposure to VR during and after the experiment. The research participants were informed that they may abort their participation at any point during the experiment without providing any explanation. All research participants were compensated with a gift card worth 99 SEK (approximately equivalent to 10 EUR) for their participation regardless of the experimental outcome.

### Driving simulator

The source code repositories for the software used in the experiment to support the simulation, collect and process data, and visualize the results are made open-source and accessible^52^ through various *DevOps* platforms. Due to the nature of the microservice software design paradigm, the software development and code-base are simplified at the cost of having higher complexity in software deployment and runtime. However, these deployment and runtime complexities are partly alleviated through exploiting containerization deployment strategy^53^. The main desktop computer handling the computations for the experiment is equipped with an intel central processing unit (11th Gen Intel(R) Core(TM) i7-11700K@3.60 GHz) and an Nvidia graphics processing unit (RTX A2000 6 GB memory, Nvidia driver version: 515) and operated by a Linux Ubuntu 22.04 LTS operating system ($\texttt {Linux kernel 5.15.0-43-generic SMP PREEMPT\_DYNAMIC}$).

#### Equipment and vehicle setup

An off-the-shelf head-mounted virtual reality headset system, HTC Vive Pro 1, was used to display the rendered simulation while capturing the head kinematics of the research participant in terms of translation and rotation. The head-mounted display (HMD) is capable of rendering an image of size 1440$\times$1600 pixel resolution per eye at 90 Hz with a $98^{\circ }$ field of view. The virtual floor height was calibrated to match the real height, approximately 1.1 m from the floor to HMD in the seated position. The HMD was further complemented with a third-party eye-tracker solution system add-on, HTC Vive Binocular Add-on, from Pupil Labs (Pupil Labs GmbH, Berlin)^54^. This device captures infra-red monochrome video feed 400$\times$400 pixel resolution per eye at 120 Hz of the eyes and pupils of the research participants. These eye data signals are then mapped as gaze points onto the head-fixed rendered image data.

There is an inherent display latency, a consistent time delay from rendering command to displaying the actual image on the device, of 0.018 s in the HTC Vive Pro 1 using low-level implementation in Python and OpenVR by other researchers^55^. However, it is unclear what operating system, drivers, and graphic rendering API were used for their experiment. Furthermore, the development of VR graphics rendering has made significant improvements to reduce the display latency using e.g. asynchronous reprojection. The reported display latency should be regarded as the worst-case display latency time due to the time of the publication. There is a similar challenge from the actuator sensor, the sensor signal latency may be found. Due to the scope and complexity of this work, these latencies will be considered negligible.

A vehicle hardware interface system is integrated to control the simulated ground vehicle. This system captures steering wheel signals for lateral control, and a pedal system set is used for longitudinal control. A *SensoWheel* steering wheel system is obtained from Sensodrive (Sensodrive GmbH, Germany) with additional mounted sensor add-ons. It can generate a fully programmable, powerful direct drive force-feedback torque towards the driver while simultaneously measuring the applied differential torque onto the steering wheel from the driver. A Logitech G27 pedal system set was repurposed for this experiment, controlling the vehicle propulsion. The vehicle hardware interface system was then mounted on top of a modified off-the-shelf racing sim cockpit (Playseat Evolution), see Fig. 4 for a view of our experimental setup with one of the authors demonstrating as a participant.

The vehicle dynamics model used in the experiment is a two-track model based on a single-seated racing vehicle as a reference with an electric propulsion system with torque vectoring applied on the rear axle. The vehicle model is paired with the *magic formula tire model* implementation^56^ with empirical parameters for racing vehicle tires. The complete vehicle model was designed and validated under *normal* driving conditions. Here the normal driving condition is characterized as $|v_y|v_x^{-1} \ll 1$ for $v_x>0$ where $v_y$ is the lateral velocity and $v_x$ is the longitudinal velocity of the vehicle. This condition allows for smaller lateral slips to be tolerated. Transient load states and extreme lateral velocity are not handled well by the model and are beyond the scope of this research project. A complete loss of control of the vehicle invalidates the trial and that trial is omitted from further analysis. The research participants are only informed that they are operating a ground vehicle but not the particular implementation or the used vehicle model prior to the experiment.

#### Calibration for gaze and video analysis in virtual reality and head-mounted displays

Several calibration routines are carried out to ensure the data is harmonized and correct. One such calibration is the lens optical correction applied in the HMD. When the rendered images are shown on the display, it is intentionally pre-distorted, e.g. chromatic aberration, in the software to compensate for the optical lenses inside the HMD. Thus it is required to account for the pre-distortion of the image coordinate system when performing gaze mapping correctly in the eye tracking software. The intrinsic calibration values used in the rendering software are stored inside the firmware loaded in the HMD systems. They are individually tuned for each HMD unit during production and may be accessed through *SteamVR* interface console. The retrieval of the correct rectilinear projection of the rendered image in the HMD is possible by applying an inverse radial distortion camera model. The numerical intrinsic camera calibration values for our particular HMD used in the experiment can be found in Supplementary Table S1.

The eye-tracking system is sensitive to slippage of the HMD, where a minor slippage may invalidate gaze data and our analysis. The 3D-model pupil detection method was chosen by default as the model is more resilient to smaller slips^57^ which limits the effects of HMD slippage to an extent. The less sophisticated 2D-model method was used as a fallback when the performance of the default 3D-model pupil detection method deteriorated. To ensure gaze data integrity, a calibration routine was performed at the beginning of the experiment for each research participant with validation routines performed at the beginning and end of the experiment. The data performance metrics for the experimental gaze data ($n=8$) were on average per person, $\text {accuracy} \approx 0.0404\,\hbox {rad}$ and $\text {precision} \approx 0.0037\,\hbox {rad}$ using the validation samples. Gaze data samples with confidence values below 60 %, associated with blinking, or classified as outliers were discarded. Here, outliers are defined as samples falling in the extreme 20 % screen border perimeter i.e. screen normalized gaze positions *x*, *y* fulfilling the conditions $x \in ]0.2,0.8[$ and $y \in ]0.2,0.8[$ are considered inliers.

#### Software architecture and systems integration for data harmonization

Various systems are used in this experiment, making the general data harmonization a complex task. The majority of data was interfaced, captured, and collected through our developed software framework *OpenDLV* using a microservice design pattern powered by the middleware software *libcluon*^53,58^. This allows for microsecond precision time stamping of the data signals when the signal enters the computer node with proper time synchronization across computer device nodes where such a feature is supported and requested. The data in the software framework is then serialized during the experimental trials and saved to multiple binary files for later processing.

The videos of the eyes of research participants and their gaze signals were collected and processed through the open-source software framework of eye-tracking system *Pupil*^54^. Our forked project has additional implementations to accommodate and process HMD-rendered image data instead of the intended head-mounted camera image feed and to account for the pre-distortion of the rendered image due to the optical lens inside of the HMD. The rendered image data in VR was captured as a *X11* window texture, then stored in an interprocess communication (IPC) shared memory allowing it to be accessed by the Pupil software for further processing.

The rendering software was developed using the open-source *OpenVR* SDK through the *Vulkan* API, an open-source low-level graphics rendering API allowing complete control of the rendering pipeline. OpenVR aims to homogenize various VR-HMD hardware systems to one common software interface API to their *SteamVR* runtime allowing VR rendering to be displayed independently of the VR-HMD. By exploiting the Vulkan API, the rendering commands of generating the frame can be captured in *gfxreconstruct* and later be used to reconstruct the frame. This is accomplished by reissuing the captured rendering commands back to the rendering pipelines, which is advantageous for later analysis stages. This method only requires a fraction of file size at a minimal resource cost during serialization compared to conventional lossless compressed video data with proper encoding. Two rendered images are produced during the experimental trials for each discrete sampled time point, one for the left eye and one for the right eye. However, for simplifying the analysis only the left-eye-rendered scene images are considered in this work.

### The experimental task in the virtual reality and data collection

**Fig. 5:**
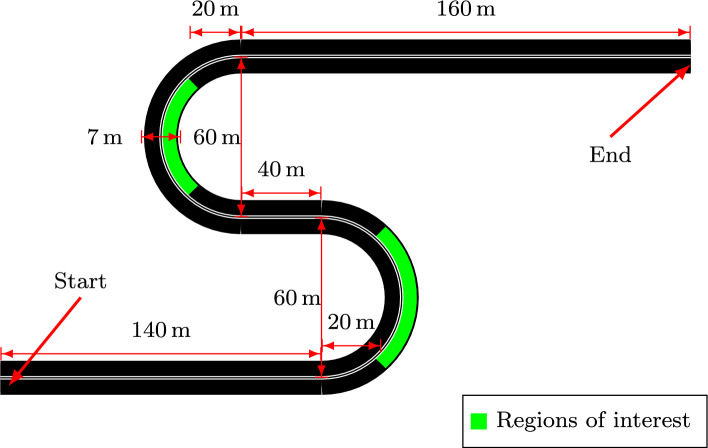
A schematic of the track design used in the experiment. The dimensions and the blueprint of an S-shaped two-lane road track are explicitly shown in Figure. The track contains straight road sections with left and right semi-circular curve bends with a diameter of 60 m. The straight road sections are created for the research participants to accelerate and decelerate to a velocity of their choice. The lanes are defined with white lines wholly drawn, with a double line in the middle separating the left and right lanes. For the analysis in this work, only the regions of interest are considered. It should be noted that the right lane in the regions of interest of left and right curve bends differ in length and radius due to being situated in the outer and inner bends.

The visual environment in VR is simplistic and kept to a minimum amount of objects: daylight outdoor scenery with blue sky and clouds, textured ground with an S-shaped two-lane road track, and a vehicle-frame-fixed gray steering wheel. The motivation for the simplistic visual environment is to make the optic flow estimation more accurate while keeping the need for post-processing at a minimum, and keeping the research participant focused on the given experimental task. The schematic details of the experiment drive track are shown in Fig. 5 and consist of an acceleration section, a left curve bend, a smaller straight section, a right curve bend, and a deceleration section. The curve bends are designed with a 60 m diameter. An example view during the left bend from the left eye of the research participant is shown in Fig. 2a.

By the design of the vehicle model, a front-axle steered vehicle traveling forward transfers a so-called *aligning torque* from the ground to the steering system, creating a stable fix point at neutral steering. The steering wheel motor used in the experiment features a direct drive force-feedback system and implements a simplistic aligning torque dynamic. Human drivers often use this aligning torque to their advantage by relaxing the control of the steering to straighten out the steering. This results in unnatural steering movements at the ends of curve bends. For this reason, these regions have been omitted from the analysis resulting in the middle sections of the curve bend (see the regions of interest in Fig. 5). These are sections where there is a considerable demand for steering maintenance and simultaneously natural steering corrections are performed.

Before the experimental task, calibration routines are carried out to ensure the integrity and accuracy of the data signals. Research participants are also asked to familiarize themselves with the VR environment and the user interfaces, allowing them to acclimate to the new visual stimuli from the HMD. This also serves as a good opportunity for them to explore all viewing angles from the driving seat as they are free to move their head during the experiment and adjust the fit of the HMD and the vehicle user interface if necessary. For the experimental task, the participants are asked to drive through the track on the right lane from start to end without a complete standstill while staying within their right lane at all times. They are also instructed to carry out the task at a comfortable and safe velocity of their preferred choice. The experiment is carried out without any external interruption or sudden intervention events. For the first five laps, the steering wheel is visible for the participants and then visibly removed for the remaining five laps, totaling ten trials for each participant. This condition was imposed to investigate how having vehicle-fixed objects in the visual vicinity may aid human locomotor performance.

Data was collected on the research participants performing the experimental task. The complete data set is divided into two: an open anonymized post-processed data set^59^ and a closed pseudonymized data set^60^ containing the pre-processed raw data with restricted access. These data sets may be accessed through the *Swedish National Data service*.

### Dense retinal optic flow estimation

The perceived visual flow in the eyes is reconstructed as numerical dense retinal optic flow emerging from relative motion to the observer. This is done by fusing optic flow estimation fixed to the head of the observers and using measured gaze to account for the rapid eye movements to reconstruct retinal optic flow. For the scope of this work, some assumptions and simplifications were made to estimate the retinal optic flow. Gazing kinematic states may be classified into four distinct types: fixation, smooth pursuit, saccade, and post-saccadic oscillation. Here, all valid active fixations will be assumed and considered as smooth pursuits, where “exact” visual gaze tracking is achieved during the fixation. Valid active fixation refers to disregard gazing data sampled during and in connection to blinking and naive outlier rejection. Exact visual gaze tracking here means that the elicited gaze point precisely follows the visual feature in the scene. This implies a pursuit gain of 1.0 relative to the visual motion which results in a null flow point at the point of gaze fixation^6–8,11,16,44^. Further, it is assumed that this occurs without major slips or catch-up saccades. For further details of this idealization of smooth pursuits, see the Supplementary Materials. This assumption allows a straightforward, simplified numerical computation of retinal optic flow using the plain naive optic flow and active gaze point data as

14 $$\begin{aligned} \varvec{v}_\text {r}(\varvec{p}, \varvec{p}_\text{ g}) = \varvec{v}( \varvec{p}) - \varvec{v}( \varvec{p}_\text{ g}) \end{aligned}$$

where $\varvec{v}_\text {r}$ is the retinal optic flow field, $\varvec{v}$ the plain naive optic flow, $\varvec{p}$ some image pixel point, and $\varvec{p}_\text{ g }$ the active gaze point in the image. One direct effect is that the optic flow vector sampled at the gaze point $\varvec{v}_\text {r}(\varvec{p}_\text{ g }, \varvec{p}_\text{ g}) = \varvec{0}$ is always the zero vector emulating the null visual flow at the fovea or exact retinal image stabilization. Then relating the angular direction of the vectors in the flow field is simply

15 $$\begin{aligned} \theta (\varvec{p}) = \arctan \frac{ v_z(\varvec{p})}{ v_y(\varvec{p})} \end{aligned}$$

where $\varvec{v} = (v_y, v_z)$ the vector components, and $\varvec{p}$ is a point sampled in the field. It should be noted that this uses the image space as the reference system, which needs to be accounted for in later analysis due to constant head tilts due to misalignment.

The dense optic flow in computer vision refers to a motion vector field sampled at every pixel in the data image frame, as opposed to sparse optic flow where only a few select points in the image describe flow vectors, refer to Supplementary Fig. S1 for visualization of synthetic examples of sparse and dense optic flow. There are a plethora of optic flow or scene flow (visual motion flow in three dimensions) estimation methods with varying accuracy and compute performance, especially since the recent introduction of black-box deep learning approaches^61–63^. Considering the data throughput, the accuracy-to-compute efficiency trade-off, and hardware-accelerated computation capabilities, *Nvidia optic flow SDK 2.0* is used in our work to estimate the optic flow. This method is accessible through the open-source computer vision library, *OpenCV*. However, its implementation is not fully disclosed and may vary depending on the generation of the Nvidia graphics card due to the optic flow estimation implementation being tied to the hardware architecture. Our source code for computing the dense retinal optic flow is made openly available^52^.

Each visual view frame of the scene from the HMD of the research participant was reconstructed and processed losslessly from the left eye. The post-rendering of the scene was carried out *without* the steering wheel to simplify the image post-processing in the analysis, such as the lane detection segmentation and optic flow estimation. The dense retinal optic flow could then be reconstructed by combining the dense optic flow and the gaze data from the eye-tracker system, see Fig. 2c for retinal optic flow field visualization.

Assuming there is no slippage of the HMD during the experiment, there is a significant constant angular tilt bias in the visually rendered image. There are many contributing factors to this, e.g. the mounted HMD is slightly misaligned or research participants naturally have their head slightly tilted during the experiment. This angular head tilt directly affects the retinal optic flow angle ${\bar{\theta }}_\text {rof}$ which is derived from the image space as the reference coordinate system. To mitigate this and make the results comparable across participants, the median of the retinal optic flow angle (constant bias) using the samples during the straight sections of the drive track is subtracted from the retinal optic flow angle samples. This re-centers the data around zero across research participants, thus making the retinal optic flow angle ${\bar{\theta }}_\text {rof} \approx 0.0$ during the straight drive sections. This angular tilt bias was small for the majority of the participants. However, some participants had a significant tilt of their view in the HMD skewing the retinal optic flow angle measures.

### Using particle swarm optimization to identify ballistic corrections through decomposition of the sum of Gaussian functions

Naturalistic limb movements, known as reachings, are composed of complex movement patterns through overlapping simpler ballistic corrections. These reaching corrections are often bell-shaped in their velocity profiles^34^ and may be approximated by Gaussian functions. Thus the general limb movements may be decomposed into their constituent corrections with varying parameters of strength $a_\text{ I }$, mode $\mu _\text{ I }$, and rate $\sigma _\text{ I }$ parameters as described in Eq. (5) and the resulting limb movement in Eq. (6).

This challenge of decomposing a sum of Gaussian functions is not specific to the domain of biomechanics. It is also found in the scientific and engineering fields of astronomy, spectroscopy, signal processing, data compression, and image processing. Thus, there are existing methods to decompose the composed signal with varying results depending on the emphasized metrics, for example, scale-space imaging using Levenberg-Marquardt algorithm^64^, deconvolution techniques^65^, and iterative *method for decomposing a sum of Gaussian functions*^66^. Since researchers from various fields have attempted to design their algorithms, they utilized varying assumptions and constraints that might only apply to their particular field. Such examples could be a priori knowing, how many constituent Gaussian functions the signal should be decomposed into, or whether the Gaussian components are strictly positive, etc.

This work developed and implemented a meta-heuristic optimization method algorithm, *particle swarm optimization* (PSO), to decompose the observed signal to its approximate individual constituent Gaussian contributions. Meta-heuristic or evolutionary algorithms are methods inspired by biological processes to solve various problems by iteratively optimizing to converge to a candidate solution to an objective or fitness function. Major advantages of using such meta-heuristic algorithms as opposed to classical optimization methods are that they generally scale better with regards to dimensions of the search space, do not need to explicitly compute the derivatives of any order of the objective function, and are comparably robust to measurement noise^67^. However, meta-heuristic optimization methods including the PSO algorithm, perform exhaustive searches in the well-defined search space and do not guarantee the candidate solution to be the global optima. Optional a priori constraints may be imposed upon the search space to induce quicker convergence and to reject false solutions.

To experimentally determine the ballistic corrections, assume the observed signal with a train of correction can be described as a sum of Gaussian functions, as described in Eq. 6, then the objective or fitness function to solve for a given time interval becomes

16 $$\begin{aligned} \Omega _{{\dot{\delta }}}^* = \mathop {\mathrm {arg\,min}}\limits _{\Omega _{{\dot{\delta }}}} \frac{N_{\dot{\delta }}^\alpha }{t_1-t_0} \int _{t_0}^{t_1} |z(t) - \sum _{(a_i,\mu _i,\sigma _i) \in \Omega _{{\dot{\delta }}}} {\dot{\delta }}_\text{ b } (t,a_i,\mu _i,\sigma _i)| \text{ d } t, \end{aligned}$$

where $\Omega _{{\dot{\delta }}}$ is a $3\times N_\delta$ matrix where each column in the matrix contains the parameters $(a_\text{ I }, \mu _\text{ I }, \sigma _\text{ I})$ for a single ballistic correction, $\alpha$ is a parameter to balance data fitting (to avoid overfit), and *z*(*t*) is the experimentally observed signal. PSO numerically solves Eq. 16 yielding a candidate solution $\Omega _{{\dot{\delta }}}^*$ which is used for the analysis. Further PSO implementation details are provided in the Supplementary Material.

## Results

A total of fourteen research participants were recruited for the experiment. Three of the fourteen research participants were part of the pilot experiment and thus excluded from this work. 110 trials were procured from the eleven research participants, of which 104 were considered successful. An example of a failed trial would be a loss of vehicular control such that $|v_y| \gg |v_x|$. The trial outcomes from the eleven participants are shown in Table 1. Only eight participants completed ten successful trials out of the possible ten. These particular eight research participants are considered for the main analysis in this work.

Some participants commented after the experiment that they experienced the trials more difficult to complete when the steering wheel was visibly removed. This is reflected in our results where unsuccessful trials occurred more often *without* the steering wheel. The common point of failure was ultimately losing control of the vehicle and swerving out of the designated lane. This is mainly due to strong rapid steering corrections applied at high speed resulting in the loss of tire traction.

### Reconstructing and quantifying retinal optic flow stimulus in curve bends

**Fig. 6:**
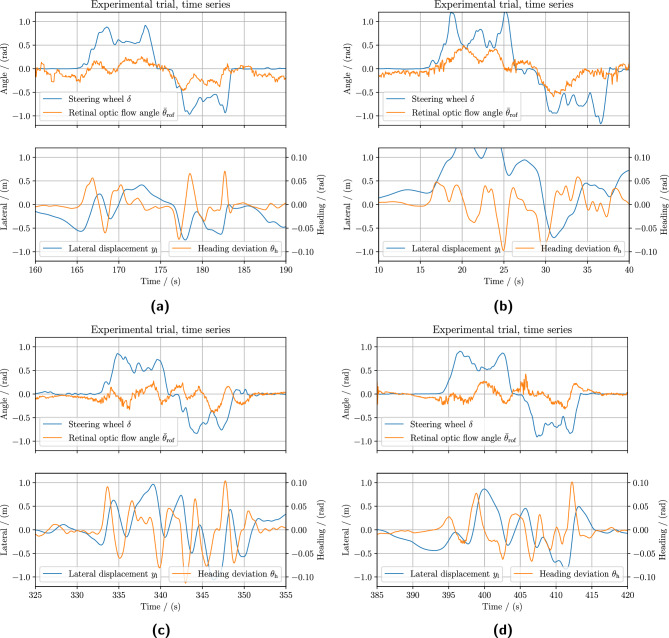
Experimental time series data for one complete trial for four research participants driving in an S-shaped track. The figure shows time series data excerpts of four different research participants driving with a visible steering wheel in panels **(a)** and **(b)**, and without a visible steering wheel in panels **(c)** and **(d)**. The upper plots in each panel show the experimental time series of the steering wheel angle $\delta$ and retinal optic flow angle $\bar{\theta }_\text {rof}$. The driving metrics vehicular lateral position $y_\text{ l }$ and vehicular heading $\theta _\text{ h }$ relative to the lane are shown in the lower plot for the same time interval. Vehicular heading deviation is related to the time derivative lateral displacement explaining its close relation. The trial consists of a left-curve bend followed by a right-curve bend, which can be identified in the steering wheel angle. Compared to the naive optic flow case, the retinal optic flow appears to be noisy but the transients are the results of the gaze fixation intermittency.

The retinal optic flow angle was computed over the lane segment region for every rendered frame, as detailed in Eq. (1) and demonstrated in Fig. 2. Figure 6 shows the time series of experimental data such as the steering wheel angle $\delta$, retinal optic flow angle ${\bar{\theta }}_\text {rof}$, heading deviation $\theta _\text{ h }$ and lateral displacement $y_\text {l}$ for one complete trial for four different research participants. An interesting observation is that the vehicular heading deviation oscillates around zero which is an essential outcome of intermittent control for successfully maintaining proper steering.

The retinal optic flow angle may look noisy at first glance. However, this is an artifact from active gaze fixation intermittency where the participant actively shifts their fixation around their driving lane within the distance headway $d_\text{ h }\in [4\,\hbox {m},25\,\hbox {m}]$ (euclidean distance to gaze point on ground from the eye participant) or the time headway $t_{{{\text{ h }}}} \in [0.4{\kern 1pt} {\text{s}},2.5{\kern 1pt} {\text{s}}]$ (distance headway normalized by the instantaneous vehicular velocity). Compared to the literature, the time headway results presented here slightly deviate partly because of a lower head position to the ground, since the participants are situated lower than in a standard passenger vehicle. Furthermore, the time headway quantity depends on the vehicular velocity, as higher velocities result in lower time headway values. Interestingly, although the research participants were free to control their longitudinal velocity on the condition they could safely execute the task, the majority still drove at a high velocity. The average velocity of $v_\text{ l } \approx {13}\,{m\,s}^{-1}$ *in the curve bends*, which corresponds to a lateral acceleration of approximately $5.63\,\hbox {m\,s}^{-2}$ (equivalent 0.57 g-force). This can partly be explained by the fact that the research participant did not have access to a speedometer during the trials. This may have resulted in them relying solely on their visual perception to subjectively misjudge their traveling speed.

**Fig. 7:**
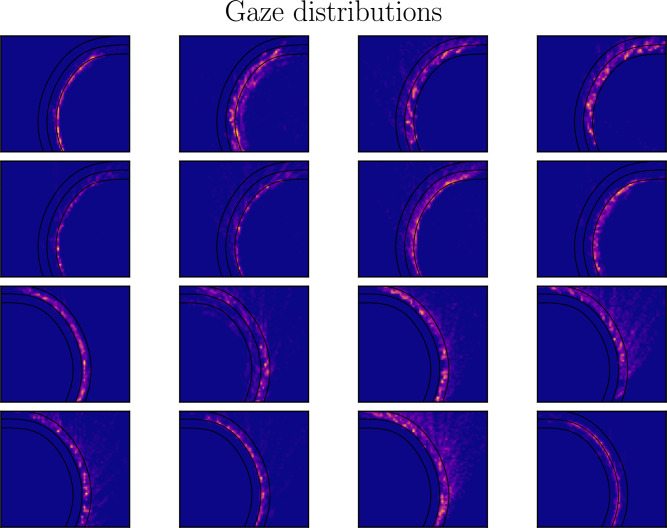
Human gaze distribution around the curve bends. The figure shows the gaze distribution as a heat map of the eight research participants for the left curve (upper rows) and right curve (lower rows). The research participants tend to fixate their gazes on the inner parts of the curve bends, close to the apex.

In the curve bends, the consistent guiding fixation behavior was emitted by the large majority of research participants. In detail, the exact placement of the guiding fixation was often placed in the neighborhood of their designated lane, more often in the inner parts of the curve around the apex line, see Fig. 7 for the gaze distribution during the curve bends. This may reinforce the idea that proper gaze control is necessary to fully exploit the retinal optic flow strategy for properly maintaining steering.

### Identifying ballistic steering corrections

**Fig. 8:**
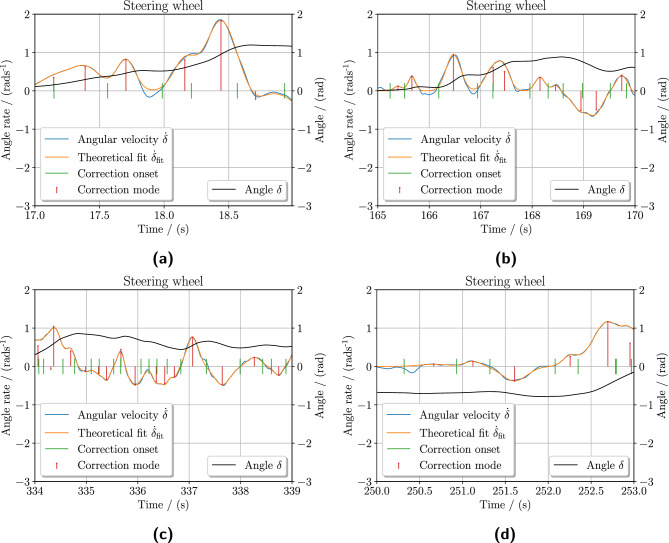
Experimentally identified intermittent ballistic corrections in the steering wheel. The steering wheel angle $\delta$ and derived angle rate ${\dot{\delta }}$ during the curve bends from the experiment are shown here for various research participants. Using particle swarm optimization, our analysis method identifies the ballistic reaching corrections ${\dot{\delta }}_\text {b}$ in the steering wheel angle rate by approximating the correction strength $a_\text{ I }$, mode $\mu _\text{ I }$, and speed rate parameter $\sigma _\text{ I }$. The stems represent the strength and mode of each correction. The resulting fitted steering wheel angle rate ${\dot{\delta }}_\text {fit}$, the sum of each theoretical ballistic correction ${\dot{\delta }}_\text {b}$, is comparable to the observed experimental data. Each correction onset timing is indicated for each ballistic correction, as defined in Eq. (8).

From the 80 trials from the eight research participants, a total of 2084 individual ballistic intermittent steering corrections were identified in the curve bends, see Table 2. Figure 8 demonstrates the identified ballistic corrections for four research participants for selected time intervals. The correction strength $a_\text{ I }$, mode $\mu _\text{ I }$, and rate $\sigma _\text{ I }$ parameters are estimated for each correction, reconstructing the resulting reaching movement as a whole using the principle of superposition as described in Eq. (6). The larger discrepancies of ${\dot{\delta }}$ and ${\dot{\delta }}_\text {fit}$ at the ends of corrections, seen in Fig. 8b at approximately 166 s and 166.8 s for an example, are most likely an effect of the aligning torque exerted as the direct drive force-feedback to the participant.

**Fig. 9:**
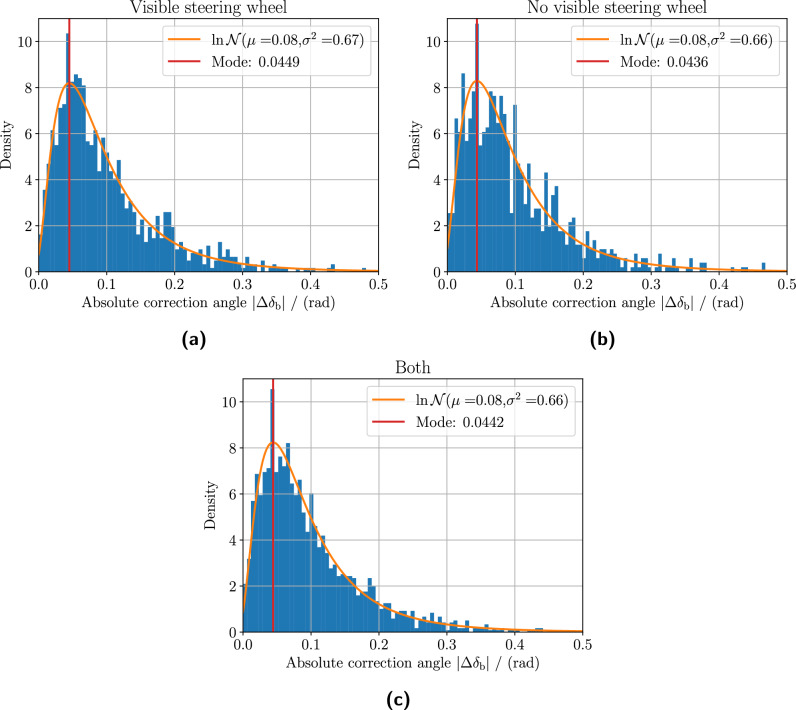
Absolute steering ballistic correction angle distributions. The figure shows the absolute steering ballistic correction angle distributions for the eight research participants who succeeded in all ten trials out of ten possible. The distribution for trials *with* visible steering wheel is shown in panel **(a)**, and the distribution for trials *without* visible steering wheel in panel **(b)**. Panel **(c)** shows the distribution using data combined from **(a)** and **(b)**. All distributions generally follow a theoretical log-normal distribution with similar parameters $\log \mathcal {N}(\mu = 0.08, \sigma ^2 = 0.66)$ in this work. Visibly removing the steering wheel does not seem to impact the ballistic steering correction angles significantly.

**Fig. 10:**
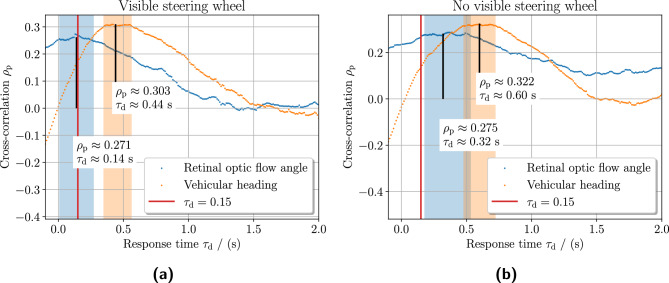
Response time analysis using zero-normalized cross-correlation. The figure shows the cross-correlation between stimuli and steering wheel correction angle over response time $\tau _\text{ d }$ for trials with a visible steering wheel in panel **(a)** and without a visible steering wheel in panel **(b)**. The cross-correlation is computed using the Pearson correlation coefficient. The response time is investigated for $\tau _{{{\text{ d }}}} \in [0{\kern 1pt} {\text{s}},2{\kern 1pt} {\text{s}}]$, measured from the ballistic steering correction onset to the stimuli onset as described in Eq. (11). The strongest cross-correlations with respect to response times for the stimuli are indicated by black vertical bars. According to the literature, the red vertical bar indicates the approximate minimum human response time directly triggered by a stimulus. The color-shaded time intervals indicate the neighborhood maximum of the cross-correlation for the two stimuli. The strongest cross-correlation between the retinal optic flow angle $\bar{\theta }_\text {rof}$ and the steering wheel correction angle $\Delta \delta _\text{ b }$ is found approximately at $\tau _\text{ d } \in \{0.14\,s,0.32\,s\}$. Equivalently for the vehicular heading $\theta _\text{ h }$, strongest cross-correlation was found $\tau _\text{ d } \in \{0.44\,s,0.60\,s\}$. Removing the visible steering wheel significantly increases the response time with approximately 0.17 s and widens the time window of the retinal optic flow angle making the steering corrections responses applied more inconsistently. There is a significant trailing tail to retinal optic flow angle when the steering wheel is visibly removed, suggesting a predictive behavior. The data views with corresponding scatter plots where the strongest cross-correlations are shown in Fig. 11.

**Fig. 11:**
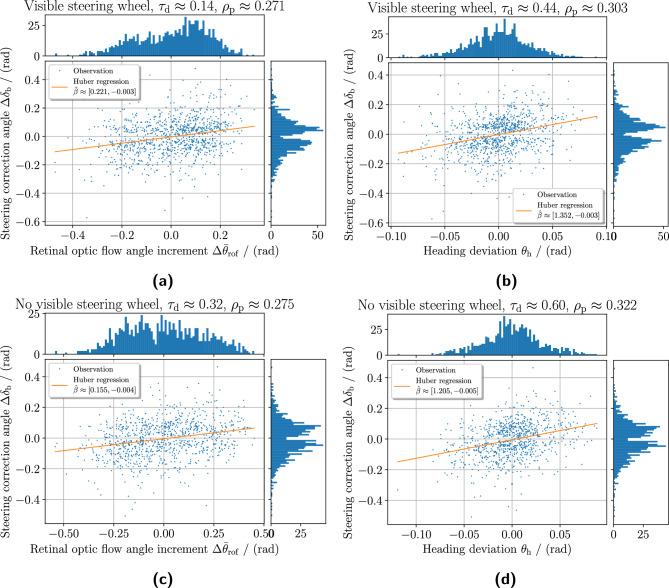
Strongest correlation at selected response time $\tau _\text{ d }$ of stimulus predictors to ballistic steering correction angle. The figure shows the relationship of the observed signals where the strongest correlation is found. Here the stimulus predictors are retinal optic flow angle and vehicle heading angle deviation, and the resulting output control signal is the ballistic steering correction angle. The panels **(a)** and **(b)** show the observation made for the successful trials with a visible steering wheel, while the panels **(c)** and **(d)** show the equivalent but without a visible steering wheel. The frequency histograms for the observed data are shown next to the scatter graphs for the respective axis. A naive Pseudo-Huber loss linear regressor is applied to the observed data approximating the linear relationship ${\hat{\varvec{\beta }}}$. The retinal optic flow angle presented here $\Delta {\bar{\theta }}_\text {rof}$ has been centered around the respective mean angle values for left and right curve bends sections to only look at the incremental values.

**Table 1:** Experiment trial outcomes.

	Visible steering wheel	No visible steering wheel	Total
Success	53	51	104
Failure	2	4	6
Total	55	55	110
Failure occurrence	3.6%	7.3%	5.5%

The table presents the trial outcomes for eleven research participants participating in five trials with a visible steering wheel and five trials *without* a visible steering wheel. Here, a success trial is defined as the participants reaching the end of the track without ever fully exiting the designated road lane. For example, excessive vehicle skidding resulting in a loss of vehicle control and sliding out of the lane would constitute a trial failure. The trial failures occurred more often without a visible steering wheel.

**Table 2:** Identified steering corrections in the curve bends.

	Visible steering wheel					No visible steering wheel					
Trial	0	1	2	3	4	5	6	7	8	9	Total
Rp 0	34	21	18	19	15	20	15	23	13	15	193
Rp 1	33	27	28	32	31	30	30	32	29	34	306
Rp 2	26	36	32	22	23	30	30	30	26	24	279
Rp 3	27	21	24	25	31	24	21	26	26	24	249
Rp 4	26	23	16	18	20	16	22	23	18	26	208
Rp 5	26	32	25	32	21	26	22	23	25	26	258
Rp 6	38	37	34	27	26	24	26	24	20	27	283
Rp 7	28	37	38	28	22	35	32	32	26	30	308
#Corrections	238	234	215	203	189	205	198	213	183	206	2084
#Trials	8	8	8	8	8	8	8	8	8	8	80
Avg. Corr./Trial	29.75	29.25	26.88	25.38	23.63	25.63	24.75	26.63	22.88	25.75	26.05
Avg.	26.98					25.13					26.05

The table presents the total number of identified corrections and the average number of corrections per trial for eight research participants with ten successful trials out of possible ten. Behavioral acclimatization or a learning effect of using repeated trials can be seen from trials 0 to 4. There is a significant trend in steering behavior where the number of corrections decreases for each lap until the fifth lap. Visibly removing the steering wheel does not significantly impact the number of corrections for each trial.

### Visually removing the steering wheel does not significantly affect the characteristics of steering corrections

The resulting trial outcomes are shown in Table 1 for the eleven research participants who participated in the experiment. The unsuccessful trials occurred more often when the steering wheel was visibly removed.

The steering behavior quantified by the number of corrections per trial lap is shown in Table 2. A clear acclimatization or learning effect can be observed in the exhibited number of steering corrections. This could be due to repeated use of identical trials or the research participant learning how to handle the vehicle better. The number of exhibited steering corrections decreases from Lap 0 to 4 (from 238 to 189). This trend cannot be observed for the remaining laps 5 to 9 with no visible steering wheel. Furthermore, visibly removing the steering wheel did not significantly impact the number of steering corrections.

Another characteristic is to look at the absolute correction angle $|\Delta \delta _\text{ b }|$ as defined in Eq. (10). The distributions of the absolute correction angle for the eight participants are shown in Fig. 9 aggregated for trials with, without a visible steering wheel, and all combined. The observed distributions follow a log-normal distribution $\log \mathcal {N}(\mu = 0.08, \sigma ^2 = 0.66)$ regardless of the visibility of the steering wheel.

### Response time of intermittent steering maintenance

So far, results of quantifying the retinal optic flow and identifying ballistic steering wheel corrections have been presented. However, the question remains if human locomotor control in curvilinear motion exploits retinal optic flow for properly maintaining steering can be observed in high-quality experimental data. Assuming that some input predictors drive output reaction responses, then every correction time onset $t_\text {I,on}$ should occur after predictor stimuli onset $t_\text {s,on}$ by a delayed response time $\tau _\text {d}$ due to cognitive processing.

Figure 10 shows the cross-correlations using the Pearson correlation coefficient $\rho _\text{ p }$ with respect to some response time $\tau _d$ for the predictor variables retinal optic flow angle $\bar{\theta }_\text {rof}$ and vehicular heading deviation $\theta _\text{ h }$ to the response variable ballistic steering wheel correction angle $\Delta \delta _b$. The human response time should be found at the strongest cross-correlation, see Eq. (11). The strongest cross-correlation between retinal optic flow angle and individual steering wheel correction angle is found at approximately $\tau _{{\text{d}}} \approx 0.14{\kern 1pt} {\text{s}}$ with visible steering wheel and $\tau _\text {d}\approx 0.32\,{\text{s}}$ without visible steering wheel respectively, see Fig. 10a and b. Equivalently for the vehicular heading deviation $\theta _\text {h}$, the strongest cross-correlation is found at approximately $\tau _\text {d}\approx 0.44\,{\text{s}}$ and $\tau _\text {d}\approx 0.60\,{\text{s}}$ respectively. From this, an additional latency of approximately 0.17 s could be observed when the steering wheel was visibly absent.

Figure 11 shows data views of the cross-correlation at the specific values of time lag $\tau _\text{ d }$ where cross-correlation was found the strongest for the predictor variables. It illustrates the observed relationship between the predictors and steering wheel correction angle $\Delta \delta _\text{ b }$ and their respective distributions. The retinal optic flow angle has been recentered around its respective mean values for left and right curve bends, thus only studying incremental angle values here. A naive Pseudo-Huber loss linear regressor has been applied to the observed data, determining the regression coefficients ${\hat{\varvec{\beta }}}$ to visualize the linear relationship better if such exists.

## Discussion

In this work, we investigated the retinal optic flow (combined with lane segmentation) and vehicular heading as a potential source of information for properly maintaining steering during curvilinear locomotion. This was done in conjunction with characterizing human lateral control as an intermittent control process where steering wheel adjustments are regarded as a ballistic correction in the form of reaching movements, with an associated strength and speed. Intermittent control makes it possible to characterize each correction with a well-defined response onset in the experimental data. A time delay analysis could be performed using cross-correlation measures. This is done by studying each ballistic control correction to retinal optic flow angle-based cues and heading deviation-based cues with respect to response time as described in Eq. (12).

The response time estimated from the retinal optic flow angle with a visible steering wheel was significantly lower than the more widely used 0.20 s. This may be explained by our definition of response time. Namely, the timestamps used for response time were measured from stimulus generation onset to correction movement onset, see Fig. 3. In this way, most of the time spent on actuating the movement is excluded from the measurement. In contrast to other experiments, it is common to include the movement travel time to the trigger of signal onset, for example, pressing a button or moving a limb above a threshold. If the movement onset in this work would instead be defined as $t_\text {s,onset} = \mu _\text{ I } - \sigma _\text{ I }$ (at 13.6 % of the correction movement) then the discrepancy difference would be $\sigma _\text{ I } \approx 0.071\,{\text{s}}$ bringing our reported values more in line with earlier studies reporting 0.20 s. This would make the response time measurements less consistent (as it would also depend on the movement travel). Recent research in mental chronometry report response timings closer to $\tau _\text {d} \approx 0.15\,{\text{s}}$ where the limb movements are excluded^21,25,29,51,68,69^. This is outlined in Fig. 11 to be used as a reference. Our definition of response time limits the influence of the reaching time on the response time, making the timings more consistent regardless of the strength and rate of the correction. One may argue onset time to be defined as $t_\text {s,onset} = \mu _\text{ I } - 3\sigma _\text{ I }$ (at 0.1 % of the correction movement) to further limit the influence of the movement on the timings effectively. However, there is also an inherent trade-off to balance; where the lower threshold is chosen, the more sensitive estimation becomes to numerical error due to computational approximation. If this is a concern, then an alternative definition may be defined as $t_\text {I,on}= \mu _\text{ I }$ at the inflection point, effectively making it independent of $\sigma _\text{ I }$.

*Spearman’s rank correlation coefficient* and *Kendall rank correlation coefficient* as an alternative measure of cross-correlation were investigated and applied in this work. The motivation is that the relationship between the stimuli predictor and the output response may not be linear, thus potentially making the Pearson correlation coefficient an unsuitable approach. Although the strength of cross-correlation varied among the methods, the general trends and shapes remained similar regardless of the method of computing the cross-correlation. For this reason, the results presented here use the Pearson correlation coefficient.

The other main finding was that visually removing the steering wheel increased the response time $\tau _\text{ d }$ of applying steering correction, further adding an approximate delay of 0.17 s which can be observed by comparing results in Fig. 10ba to Fig. 10b. At the same time, it did not significantly impact the steering correction characteristics in terms of the number of corrections or the ballistic correction angle length $|\Delta \delta _\text{ b }|$ as seen in Fig. 9. This suggests that having multiple modes to perceive different aspects of egomotion may improve locomotor performance, in this case, the timeliness (lower latency) of the applied steering correction.

There is an identifiable retinal optic flow angle peak in Fig. 10a suggested and characterized by online control. This is the definition of online-driven control where the informed control adjustments are based on readily available information. On the other hand, for model-based control, anticipatory responses are challenging to study in experiments with human participants, especially when the flow of information is continuous. Anticipatory responses are based on some previous stimuli information (possibly from other sources) combined with some internal models producing the predictive control. Implied by this, these have no typical control characteristics than a non-trivial contribution $\tau _\text{ c } > 0$. This could explain the heavy tail observed in the response time for the retinal topic flow angle seen in Fig. 10b and the larger time window when the steering wheel was visibly absent.

Regarding the debate on what perceptual cues may be exploited for maintaining steering in curvilinear paths, our results suggest significantly different time regimes for retinal optic flow angle and heading respectively. This is under the premise that these perceptual cues drive the perceived need to produce a locomotor correction response to achieve the task. There is also the possibility that these perceptual cues are simultaneously used for locomotor control. Implied by the results, suggests that retinal optic flow may operate on a closer time horizon compared to that of heading. Effectively, this makes corrections based on retinal optic flow more suitable for transients, while corrections based on heading deviation are for relatively long-term steering maintenance.

Our implementation of reconstructing the retinal optic flow and visually analyzing the lane segmentation shares similarities with other computational perception-driven human driver models, such as the *two-point control model*^70^. In short, the two-point control model samples a *near-point* to control the lateral positioning and a *far-point* for heading control. Retinal optic flow angle achieves similar results but through a different method of sampling information. The lateral displacement directly affects the lane placement in the visual periphery, which in turn dominantly affects the sampling set $\Omega _\text {lane}$ determining the retinal optic flow angle, see Fig. 2b. In our particular implementation, the samples are equally weighted in the computation of the retinal optic flow angle. However, these may be weighted non-uniformly to emphasize different parts of the lane segment to achieve better driving performance or emulate other known biological features.

Visibly removing the steering wheel significantly impacted locomotor performance when looking at the response timings. However, the number of applied steering corrections and the distribution of correction travel angle $|\Delta \delta _\text{ b }|$ remained unaffected by the change. However, the response time was increased $\tau _\text{ d }: 0.14\,{\text{s}} \rightarrow 0.32\,{\text{s}}$ for the retinal optic flow angle and $\tau _\text{ d }: 0.44\,{\text{s}} \rightarrow 0.60\,{\text{s}}$ for vehicular heading deviation (roughly an added latency of 0.17 s). This is partially supported by the comments made by the research participants after the trials, stating that they experienced increased difficulty in completing the trials when they could no longer see the steering wheel. However, it remains unclear what of the following underlying mechanisms contribute to the delayed response: (a) the direct visual feedback of the immediately applied steering correction, (b) having the steering wheel visibly rotating with the real one in aiding the perceived steering or heading direction, (c) perceiving the position of the steering wheel object aiding the perceived lateral position relative to the lane, or even (d) a combination of these. One possible hypothesis is that humans utilize the extra source of information to reaffirm internal models and by extension the ego-state estimation. Having additional modes of perceptual cues could allow for a tighter, timelier, and more accurate intermittent control correction, resulting in improved performance of steering maintenance.

Our experimental findings and interpretations are coherent with the computational framework of human driver behavior for maintaining lateral steering in curvilinear paths^29^. This is however not the case during the curve negotiations when entering and exiting the curve bends. Our cross-correlation measures for predictors to control correction rapidly diminish when including the entrance and exit regions of the curve bends, see Fig. 5. The model using retinal optic flow angle as a primary source for a universal steering control is insufficient. This might suggest different perception-control mechanisms may be at play in these contexts, e.g. predictive control or retrieval of heading, thus these driving regions should be studied separately.

Our contribution demonstrates the convergence and harmony of different theories made in isolation from various research fields. In particular, contributions from visual perception and cognitive information processing to muscle coordination and motor control in humans have been utilized, as well as contributions from engineering fields such as computer vision and signal processing. Here it is shown that using a unified model to describe human locomotor behavior is possible.

### Human locomotor behavior as a sustained sensorimotor control with intermittent responses

Our work emphasizes and demonstrates that human steering behavior is an intermittent process where humans intermittently apply incremental ballistic steering corrections to maintain their path. Although the idea is not novel^27–29^, this study makes full use of intermittent control to describe the ballistic action response. This allows us to study the human locomotor behavior as a closed loop from visually perceived stimuli to a ballistic action response

Researchers have long debated whether human behavior in a control context can be described as either strong online or model-based^18^, and further what and how perceptual information is used for the control mechanism^12^. Human behavior is a complex convoluted system with many different intermittent, concurrent, and asynchronous interplaying cognitive processes. One example of this is that human drivers are not only tasked to maintain sufficient lateral control of the vehicle but also to plan the coming long-term navigational decisions, maintain proper longitudinal velocity, and be aware or alerted to the surroundings. This is further reinforced by the fact that gazing itself during driving does not always lead to lateral control^71^. We argue that the human chaotic gazing behavior is an attentive *monitoring* process to intermittently sample relevant information in order to judge the need to correct, a *satisficing* behavior.

Our results may provide additional value and insight to the debate of human sensorimotor control as a strong online-driven or model-based^18^. Previous research findings for a strong online-driven control^21,29^ such that responses approaches $\tau _\text{ d } \approx 0.15\,{\text{s}}$. Furthermore, it is implied that humans complete the muscle control process before the neurocognitive processes of attention, planning, and prediction are fully completed. One interpretation of this phenomenon is that humans are not completely aware of their actions until the action itself has been carried out^24,72,73^. Another interpretation is that the control is both reactive and corrective. An initial correction response is first issued, and if it is insufficient or does not match the intended anticipated outcome, additional corrections are applied, as proposed in the other works^29^. This offers an explanation for the observed trains of overlapping corrections in the steering wheel, see Fig. 8c for example.

In our results, when the steering wheel was visibly absent, the participants may have produced anticipatory responses to maintain their curvilinear paths. This strategy may have been a coping strategy as the participants lost a reliable source of visual information used to update and monitor their steering corrections. This may explain the observable increase in the response time in our results. It has been previously shown that humans use predictive gaze strategies in the driving context when presented with predictable patterns of *waypoints* to sample information for the next course of action^17^. Similarly, human perception tends to find patterns to make *informed* predictive guesses to carry out actions appropriately^74^. For humans, forming cognitive internal models seems to be a central component of visualizing, achieving, and carrying out the task through consequential analysis; *to accomplish that, this needs to be done*. This process could be an evolutionary artifact to condition human behavior to successful and desirable outcomes based on memories and past experiences.

Yet, at the same time, it would seem that cognitively humans initiate actions before the planning and prediction processes are completed, or surprisingly are fully aware of their actions themselves. It is yet unclear how exactly humans can instinctively and hastily perform the initial response without having a complete plan and fully mapped actions to execute. One hypothesis is that the response itself is not required to be critically correct, but the emphasis on its timeliness with *sufficient* accuracy of the rapid corrections^22,23,25^. Would the rapid response not lead to the desirable outcome or align with the internalized prediction model, through a process called neuronal evidence accumulation^29^, an *additional* incremental correctional responses are applied on the fly. This process may also make use of immediate available perceptual information to quickly update internal models of which to enact a newly (re)weighted response to achieve the task^3,25^.

In summary, human locomotor control should be understood as a hybrid control with intermittent correction responses making use of both immediately readily available information and internal models to account for anticipation and prediction to monitor previously applied corrections. This enables the human to internalize the most favorable outcome in addition to *monitor* and continuously evaluate the self-perceived ego-state. Furthermore, human control modeling should be analyzed and viewed as an intermittent control process, where ballistic corrections are continuously and asynchronously applied to steer it to a targeted goal.

## Conclusions

This work investigated how retinal optic flow and direction can serve as a perception cue to maintain steering for curvilinear paths. Visually rendered images and vehicular data were collected from eight adult research participants driving in an S-shaped drive track in a texture-rich environment in virtual reality. In data analysis, the retinal optic flow was reconstructed with the help of an eye-tracking system that accounts for the gaze dynamics. Retinal optic flow and lane segmentation, from the readily available visual environment, enabled a perception quantity of angular directional for further analysis for human locomotor control in curvilinear paths. The lateral control during the curve bends exhibited by the research participants was studied as intermittent ballistic reaching movements, similar to the naturalistic reaching movements in limbs. Each ballistic steering wheel correction was identified using PSO to estimate the correction mode, the correction rate parameter, and the correction strength. The impact on driving performance when having a vehicle-fixed steering wheel visible was also investigated.

The data, from the eight research participants who successfully completed ten trials out of ten possible, reveal significant cross-correlational measures between directional ballistic steering correction angles for retinal optical flow-based and heading-based stimuli at different response time regimes. It was previously known that emerging human gaze behavior achieves retinal image stabilization. However, we argue through our findings that it has applications beyond solely image stabilization, for human locomotor control in curvilinear motion. The placement of the gaze and the fixation provides an additional mode to perceive the need for control or evidence accumulation. Furthermore, under normal driving conditions with a visible steering wheel, the time delay analysis reveals that the correlation between the retinal optic flow-based cue and the steering correction response is the strongest at approximately 0.14 s from the onset of sensation to the onset of movement. Simultaneously for the heading-based stimuli cue, the response time is found at 0.44 s (compared to the reported 0.15 s response time according to other literature).

It was also found that hiding the vehicle-fixed steering wheel significantly delayed the initiation of the ballistic steering correction. This delay was observed to be approximately 0.17 s. However, the absence of a visible steering wheel did not substantially affect the characteristics of the corrections in the steering wheel: the number of corrections or the angle length of the steering correction.

The consistency in response time suggests an online control for steering maintenance during curvilinear paths. At the same time, when visibly removing the steering wheel indicates reliance on predictive control or anticipatory responses for the retinal optic flow-based cue, arguing for model-based control may be at play. This is in line with the sustained sensorimotor control model with intermittent decisions of human locomotor behavior, which is a hybrid approach using both online control and model-based control. Thus, human locomotor control behavior should be understood as a system with various overlapping cooperative and coordinated cognitive processes that ultimately accomplish a task through a collaborative and collective effort.

## Supplementary Information

Supplementary Information.

## Data Availability

The complete data set consisting of raw and analyzed data has been divided into two. The open data set contains the post-processed anonymized data, which is made openly available through the Swedish National Data service^59^. The restricted closed data set containing raw sensitive and pseudonymized personal data may be granted access upon reasonable request through the Swedish National Data service^60^. Example videos demonstrating the experiment and data visualization of the author driving can be found here: https://youtu.be/rW_X9OZ3mRM and https://youtu.be/d0xtk2d4wFk (with GUI).

## References

[1] Zeil, J. Visual navigation: properties, acquisition and use of views. *J. Comp. Physiol. A* **209**, 499–514. 10.1007/s00359-022-01599-2 (2023). 10.1007/s00359-022-01599-2 36515743

[2] Avitan, L. et al. Spontaneous activity in the zebrafish tectum reorganizes over development and is influenced by visual experience. *Curr. Biol.* **27**, 2407-2419.e4. 10.1016/j.cub.2017.06.056 (2017). 28781054 10.1016/j.cub.2017.06.056

[3] Madhav, M. S. et al. Control and recalibration of path integration in place cells using optic flow. *Nature Neurosci.*10.1038/s41593-024-01681-9 (2024). 38937582 10.1038/s41593-024-01681-9 PMC11563580

[4] Matthis, J. S., Yates, J. L. & Hayhoe, M. M. Gaze and the control of foot placement when walking in natural terrain. *Curr. Biol.* **28**, 1224-1233.e5. 10.1016/j.cub.2018.03.008 (2018). 29657116 10.1016/j.cub.2018.03.008 PMC5937949

[5] Barton, S. L., Matthis, J. S. & Fajen, B. R. Control strategies for rapid, visually guided adjustments of the foot during continuous walking. *Exp. Brain Res.* **237**, 1673–1690. 10.1007/s00221-019-05538-7 (2019). 30976822 10.1007/s00221-019-05538-7

[6] Kim, N.-G. & Turvey, M. Eye movements and a rule for perceiving direction of heading. *Ecol. Psychol.* **11**, 233–248. 10.1207/s15326969eco1103_3 (1999).

[7] Wann, J. P. & Swapp, D. K. Why you should look where you are going. *Nature Neurosci.* **3**, 647–648. 10.1038/76602 (2000). 10862695 10.1038/76602

[8] Raudies, F., Mingolla, E. & Neumann, H. Active gaze control improves optic flow-based segmentation and steering. *PLOS ONE* **7**, 1–19. 10.1371/journal.pone.0038446 (2012). 10.1371/journal.pone.0038446 PMC3375264 22719889

[9] Wilkie, R. M. & Wann, J. P. Eye-movements aid the control of locomotion. *J. Vis.* **3**, 3–3. 10.1167/3.11.3 (2003). 10.1167/3.11.3 14765952

[10] Matthis, J. S., Muller, K. S. & Hayhoe, M. M. Retinal optic flow and the control of locomotion. *J. Vis.* **19**, 179–179. 10.1167/19.10.179 (2019).

[11] Matthis, J. S., Muller, K. S., Bonnen, K. L. & Hayhoe, M. M. Retinal optic flow during natural locomotion. *PLOS Comput. Biol.* **18**, 1–37. 10.1371/journal.pcbi.1009575 (2022). 10.1371/journal.pcbi.1009575 PMC8896712 35192614

[12] Wann, J. & Land, M. Steering with or without the flow: is the retrieval of heading necessary?. *Trends Cognit. Sci.* **4**, 319–324. 10.1016/S1364-6613(00)01513-8 (2000). 10904256 10.1016/s1364-6613(00)01513-8

[13] Wann, J. P. & Wilkie, R. M. How do we control high speed steering? In Vaina, L. M., Beardsley, S. A. & Rushton, S. K. (eds.) *Optic Flow and Beyond*, 401–419, 10.1007/978-1-4020-2092-6_18 (Springer Netherlands, Dordrecht, 2004).

[14] Henderson, J. M. Human gaze control during real-world scene perception. *Trends Cognit. Sci.* **7**, 498–504. 10.1016/j.tics.2003.09.006 (2003). 14585447 10.1016/j.tics.2003.09.006

[15] Esko Lehtonen, H. K., Lappi, O. & Summala, H. Look-ahead fixations in curve driving. *Ergonomics* **56**, 34–44. 10.1080/00140139.2012.739205 (2013). 23140361 10.1080/00140139.2012.739205

[16] Lappi, O. et al. Humans use optokinetic eye movements to track waypoints for steering. *Sci. Rep.* **10**, 4175. 10.1038/s41598-020-60531-3 (2020). 32144287 10.1038/s41598-020-60531-3 PMC7060325

[17] Tuhkanen, S. et al. Humans use predictive gaze strategies to target waypoints for steering. *Sci. Rep.* **9**, 1–18. 10.1038/s41598-019-44723-0 (2019). 31171850 10.1038/s41598-019-44723-0 PMC6554351

[18] Zhao, H. & Warren, W. H. On-line and model-based approaches to the visual control of action. *Vis. Res.* **110**, 190–202. 10.1016/j.visres.2014.10.008 (2015). 25454700 10.1016/j.visres.2014.10.008 PMC4404165

[19] Henderson, J. M. Gaze control as prediction. *Trends Cognit. Sci.* **21**, 15–23. 10.1016/j.tics.2016.11.003 (2017). 27931846 10.1016/j.tics.2016.11.003

[20] Hayhoe, M. M., McKinney, T., Chajka, K. & Pelz, J. B. Predictive eye movements in natural vision. *Exp. Brain Res.* **217**, 125–136. 10.1007/s00221-011-2979-2 (2012). 22183755 10.1007/s00221-011-2979-2 PMC3328199

[21] Perfiliev, S., Isa, T., Johnels, B., Steg, G. & Wessberg, J. Reflexive limb selection and control of reach direction to moving targets in cats, monkeys, and humans. *J. Neurophysiol.* **104**, 2423–2432. 10.1152/jn.01133.2009 (2010). 20810693 10.1152/jn.01133.2009

[22] Whitney, D., Westwood, D. A. & Goodale, M. A. The influence of visual motion on fast reaching movements to a stationary object. *Nature* **423**, 869–873. 10.1038/nature01693 (2003). 12815432 10.1038/nature01693 PMC3890253

[23] Gosselin-Kessiby, N., Messier, J. & Kalaska, J. F. Evidence for automatic on-line adjustments of hand orientation during natural reaching movements to stationary targets. *J. Neurophysiol.* **99**, 1653–1671. 10.1152/jn.00980.2007 (2008). 18256170 10.1152/jn.00980.2007

[24] Yeom, H. G., Kim, J. S. & Chung, C. K. Brain mechanisms in motor control during reaching movements: transition of functional connectivity according to movement states. *Sci. Rep.* **10**, 567. 10.1038/s41598-020-57489-7 (2020). 31953515 10.1038/s41598-020-57489-7 PMC6969071

[25] Gallivan, J. P., Chapman, C. S., Wolpert, D. M. & Flanagan, J. R. Decision-making in sensorimotor control. *Nature Rev. Neurosci.* **19**, 519–534. 10.1038/s41583-018-0045-9 (2018). 30089888 10.1038/s41583-018-0045-9 PMC6107066

[26] Vince, M. The intermittency of control movements and the psychological refractory period. The British journal of psychology. *General Sect.* **38**, 149–157. 10.1111/j.2044-8295.1948.tb01150.x (1948). 10.1111/j.2044-8295.1948.tb01150.x 18913658

[27] Craik, K. J. W. Theory of the human operator in control systems. *British J. Psychol. General Sect.* **38**, 56–61. 10.1111/j.2044-8295.1947.tb01141.x (1947). 10.1111/j.2044-8295.1947.tb01141.x 18917476

[28] Benderius, O. & Markkula, G. Evidence for a fundamental property of steering. *Proce. Human Factors Ergon. Soc. Ann. Meet.* **58**, 884–888. 10.1177/1541931214581186 (2014).

[29] Markkula, G., Boer, E., Romano, R. & Merat, N. Sustained sensorimotor control as intermittent decisions about prediction errors: computational framework and application to ground vehicle steering. *Biol. Cybernet.* **112**, 181–207. 10.1038/s41598-019-44723-0 (2018). 10.1007/s00422-017-0743-9 PMC6002515 29453689

[30] Tuhkanen, S., Pekkanen, J., Mole, C., Wilkie, R. M. & Lappi, O. Can gaze control steering?. *J. Vis.* **23**, 12–12. 10.1167/jov.23.7.12 (2023). 37477935 10.1167/jov.23.7.12 PMC10365140

[31] Gawthrop, P., Loram, I., Lakie, M. & Gollee, H. Intermittent control: a computational theory of human control. *Biol. Cybernet.* **104**, 31–51. 10.1007/s00422-010-0416-4 (2011). 10.1007/s00422-010-0416-4 21327829

[32] Gielen, S. C. A. M. *Motor Control Models*, 2428–2431. 10.1007/978-3-540-29678-2_3584.

[33] Binder, M. D., Hirokawa, N. & Windhorst, U. (eds.). *Bell-shaped Speed Profile*, 375–375.10.1007/978-3-540-29678-2_599.

[34] Plamondon, R. On the origin of asymmetric bell-shaped velocity profiles in rapid-aimed movements. In Requin, J. & Stelmach, G. E. (eds.) *Tutorials in Motor Neuroscience*, 283–295,10.1007/978-94-011-3626-6_23 (Springer Netherlands, Dordrecht, 1991).

[35] D’avella, A. & Lacquaniti, F. Control of reaching movements by muscle synergy combinations. *Front. Comput. Neurosci.*10.3389/fncom.2013.00042 (2013). 23626534 10.3389/fncom.2013.00042 PMC3630368

[36] Bizzi, E. & Cheung, V. C. The neural origin of muscle synergies. *Front. Comput. Neurosci.*10.3389/fncom.2013.00051 (2013). 23641212 10.3389/fncom.2013.00051 PMC3638124

[37] Cheung, V. C. et al. Plasticity of muscle synergies through fractionation and merging during development and training of human runners. *Nature Commun.* **11**, 4356 (2020). 32868777 10.1038/s41467-020-18210-4 PMC7459346

[38] Dominici, N. et al. Locomotor primitives in newborn babies and their development. *Science* **334**, 997–999. 10.1038/s41467-020-18210-4 (2011). 22096202 10.1126/science.1210617

[39] Folina, J. Big ideas: the power of a unifying concept. *J. Gen. Philos. Sci.* **54**, 149–168. 10.1007/s10838-022-09628-z (2023).

[40] Kao, M. Unification leyond justification: a strategy for theory development. *Synthese* **196**, 3263–3278. 10.1007/s11229-017-1515-8 (2019).

[41] Niehorster, D. C., Cheng, J. C. K. & Li, L. Optimal combination of form and motion cues in human heading perception. *J. Vis.* **10**, 20–20. 10.1167/10.11.20 (2010). 20884515 10.1167/10.11.20

[42] Li, L., Sweet, B. T. & Stone, L. S. Humans can perceive heading without visual path information. *J. Vis.* **6**, 2–2. 10.1167/6.9.2 (2006). 10.1167/6.9.2 17083281

[43] Crowell, J. A., Banks, M. S., Shenoy, K. V. & Andersen, R. A. Visual self-motion perception during head turns. *Nature Neurosci.* **1**, 732–737. 10.1038/3732 (1998). 10196591 10.1038/3732

[44] Wann, J. P. & Swapp, D. K. Supplementary appendix: Why you should look where you are going. *Nature Neurosci.* **3**, 647–648. 10.1038/76602 (2000). 10862695 10.1038/76602

[45] Binder, M. D., Hirokawa, N. & Windhorst, U. (eds.). *Point-to-Point movements*, 3173–3173. 10.1007/978-3-540-29678-2_4627.

[46] d’Avella, A. *Reaching movements*, 3363–3367.10.1007/978-3-540-29678-2_4936.

[47] Grimme, B., Lipinski, J. & Schöner, G. Naturalistic arm movements during obstacle avoidance in 3d and the identification of movement primitives. *Exp. Brain Res.* **222**, 185–200. 10.1007/s00221-012-3205-6 (2012). 22996050 10.1007/s00221-012-3205-6

[48] Cook, E. P. & Maunsell, J. H. Dynamics of neuronal responses in macaque mt and vip during motion detection. *Nature Neurosci.* **5**, 985–994. 10.1038/nn924 (2002). 12244324 10.1038/nn924

[49] Purcell, B. A. et al. Neurally constrained modeling of perceptual decision making. *Psychol. Rev.* **117**, 1113. 10.1037/a0020311 (2010). 20822291 10.1037/a0020311 PMC2979343

[50] Lamarre, Y., Spidalieri, G. & Lund, J. P. Patterns of muscular and motor cortical activity during a simple arm movement in the monkey. *Can. J. Physiol. Pharmacol.* **59**, 748–756. 10.1139/y81-111 (1981) (**PMID: 7317854**). 7317854 10.1139/y81-111

[51] Morrow, M. M. & Miller, L. E. Prediction of muscle activity by populations of sequentially recorded primary motor cortex neurons. *J. Neurophysiol.* **89**, 2279–2288. 10.1152/jn.00632.2002 (2003) (**PMID: 12612022**). 12612022 10.1152/jn.00632.2002 PMC2586069

[52] Nguyen, B. & Benderius, O. Source code for: Intermittent control and retinal optic flow when maintaining a curvilinear path. *Zenodo* (2024). 10.5281/zenodo.13683462 PMC12122885 40442194

[53] Berger, C., Nguyen, B. & Benderius, O. Containerized development and microservices for self-driving vehicles: Experiences & best practices. In *2017 IEEE International Conference on Software Architecture Workshops (ICSAW)*, 7–12, 10.1109/ICSAW.2017.56 (2017).

[54] Kassner, M., Patera, W. & Bulling, A. Pupil: An open source platform for pervasive eye tracking and mobile gaze-based interaction. In *Adjunct Proceedings of the 2014 ACM International Joint Conference on Pervasive and Ubiquitous Computing*, UbiComp ’14 Adjunct, 1151–1160, 10.1145/2638728.2641695 (ACM, New York, NY, USA, 2014).

[55] Le Chénéchal, M. & Chatel-Goldman, J. HTC Vive Pro time performance benchmark for scientific research. In *ICAT-EGVE 2018* (Limassol, Cyprus, 2018).

[56] Pacejka, H. B. & Bakker, E. The magic formula tyre model. *Veh. Syst. Dyn.* **21**, 1–18. 10.1080/00423119208969994 (1992).

[57] Dierkes, K., Kassner, M. & Bulling, A. A fast approach to refraction-aware eye-model fitting and gaze prediction. In *Proceedings of the 11th ACM Symposium on Eye Tracking Research & Applications*, ETRA ’19, 10.1145/3314111.3319819 (Association for Computing Machinery, New York, NY, USA, 2019).

[58] Nguyen, B., Berger, C. & Benderius, O. Systematic benchmarking for reproducibility of computer vision algorithms for real-time systems: The example of optic flow estimation. In *2019 IEEE/RSJ International Conference on Intelligent Robots and Systems (IROS)*, 5264–5269, 10.1109/IROS40897.2019.8968066 (2019).

[59] Nguyen, B. & Benderius, O. Open data set for: Intermittent control and retinal optic flow when maintaining a curvilinear path. *Swedish National Data Service* (2024). 10.5878/vjkp-z436 PMC12122885 40442194

[60] Nguyen, B. & Benderius, O. Closed raw data set for: Intermittent control and retinal optic flow when maintaining a curvilinear path. *Swedish National Data Service* (2024). 10.5878/xnax-zk75 PMC12122885 40442194

[61] Baker, S. et al. A database and evaluation methodology for optical flow. *Int. J. Comput. Vis.* **92**, 1–31. 10.1007/s11263-010-0390-2 (2011).

[62] Geiger, A., Lenz, P. & Urtasun, R. Are we ready for autonomous driving? the kitti vision benchmark suite. In *2012 IEEE Conference on Computer Vision and Pattern Recognition*, 3354–3361, 10.1109/CVPR.2012.6248074 (2012).

[63] Menze, M. & Geiger, A. Object scene flow for autonomous vehicles. In *2015 IEEE Conference on Computer Vision and Pattern Recognition (CVPR)*, 3061–3070, 10.1109/CVPR.2015.7298925 (2015).

[64] Goshtasby, A. & Oneill, W. Curve fitting by a sum of gaussians. *CVGIP Graph. Models Image Process.* **56**, 281–288. 10.1006/cgip.1994.1025 (1994).

[65] Monnet, G., Bacon, R. & Emsellem, E. Modelling the stellar intensity and radial velocity fields in triaxial galaxies by sums of Gaussian functions. *Astronomy Astrophys.* **253**, 366–373 (1992).

[66] Kovalenko, A., Vovk, S. & Plakhtii, Y. G. Sum decomposition method for gaussian functions comprising an experimental photoluminescence spectrum. *J. Appl. Spectroscopy* **88**, 357–362. 10.1007/s10812-021-01182-8 (2021).

[67] Wahde, M. *Biologically inspired optimization methods: an introduction* (WIT press, 2008).

[68] Nash, C. J., Cole, D. J. & Bigler, R. S. A review of human sensory dynamics for application to models of driver steering and speed control. *Biol. Cybernet.* **110**, 91–116. 10.1007/s00422-016-0682-x (2016). 10.1007/s00422-016-0682-x PMC4903114 27086133

[69] Selen, L. P., Shadlen, M. N. & Wolpert, D. M. Deliberation in the motor system: reflex gains track evolving evidence leading to a decision. *J. Neurosci.* **32**, 2276–2286. 10.1523/jneurosci.5273-11.2012 (2012). 22396403 10.1523/JNEUROSCI.5273-11.2012 PMC3299561

[70] Salvucci, D. D. & Gray, R. A two-point visual control model of steering. *Perception* **33**, 1233–1248. 10.1068/p5343 (2004) (**PMID: 15693668**). 15693668 10.1068/p5343

[71] Lehtonen, E. et al. Gaze doesn’t always lead steering. *Accident Anal. Prevent.* **121**, 268–278. 10.1016/j.aap.2018.09.026 (2018). 10.1016/j.aap.2018.09.026 30292866

[72] Libet, B. Unconscious cerebral initiative and the role of conscious will in voluntary action. *Behav. Brain Sci.* **8**, 529–539. 10.1017/S0140525X00044903 (1985).

[73] Matsuhashi, M. & Hallett, M. The timing of the conscious intention to move. *Eur. J. Neurosci.* **28**, 2344–2351. 10.1111/j.1460-9568.2008.06525.x (2008). 19046374 10.1111/j.1460-9568.2008.06525.x PMC4747633

[74] Enns, J. T. & Lleras, A. What’s next? new evidence for prediction in human vision. *Trends Cognit. Sci* **12**, 327–333. 10.1016/j.tics.2008.06.001 (2008). 18684660 10.1016/j.tics.2008.06.001

