# Supplementary material for: Intermittent control and retinal optic flow when maintaining a curvilinear path
Source: Sci Rep. 2025 May 29;15:18926. doi: 10.1038/s41598-025-02402-3 (PMC12122885; doi:10.1038/s41598-025-02402-3)
Supplement: Supplementary file 1 — Supplementary Information. [file 41598_2025_2402_MOESM1_ESM.pdf]

Intermittent control and retinal optic flow when maintaining a curvilinear path

Björnborg Nguyen[1,*] and Ola Benderius[1,+]

[1]Chalmers University of Technology, Department of Mechanics and Maritime Sciences, 412 96 Göteborg, Sweden
[*]bjornborg.nguyen@chalmers.se
[+]ola.benderius@chalmers.se

Figures

Visualization of optic flow

Optic flow can be considered as a two-dimensional vector field. Traditionally, a quiver representation may be intuitive and sufficient when conveying the general flow field dynamics but is limited in how the arrows are sampled. An alternative to visualize the vector field is to utilize a color-encoding to present the direction and magnitude of the field. Here a hue-saturation-value (HSV) lossy encoding is employed where the field direction is presented by the color hue, and the magnitude by color saturation. Figure S1 illustrates theoretical examples of divergence, curl, translation, and combination of all former examples for the sparse and dense vector fields. The dense optic flow visualization is made symmetric around the vertical axis to simplify the visualization, i.e. vectors pointing leftward and rightward are colored the same (red).

Theoretical reaching movements

The main article outlines that the velocity profile of a ballistic reaching correction may be approximated through a theoretical Gaussian function. For completeness, the acceleration profile of the ballistic correction is simply the time derivative of Eq. (5)

$$\ddot{\delta}_{\mathrm{b}}(t, a_{\mathrm{I}}, \mu_{\mathrm{I}}, \sigma_{\mathrm{I}}) = \frac{\mathrm{d}}{\mathrm{d}t} a_{\mathrm{I}} \exp \frac{-(t-\mu_{\mathrm{I}})^2}{2\sigma_{\mathrm{I}}^2} = \frac{a_{\mathrm{I}}(\mu_{\mathrm{I}} - t)}{\sigma_{\mathrm{I}}^2} \exp \frac{-(t-\mu_{\mathrm{I}})^2}{2\sigma_{\mathrm{I}}^2}. \tag{S1}$$

The theoretical position, velocity, and acceleration profiles as presented in this work for a single ballistic correction and complex overlapping ballistic corrections are illustrated in Fig. S2.

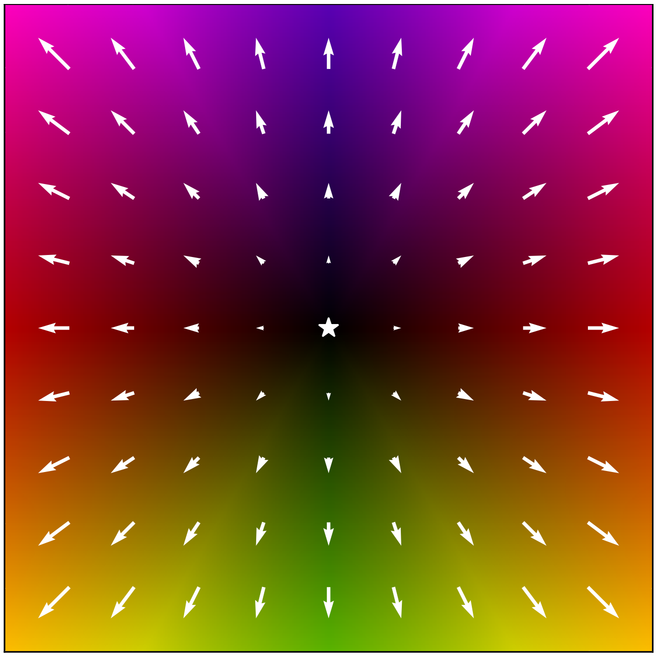

(a) Divergence

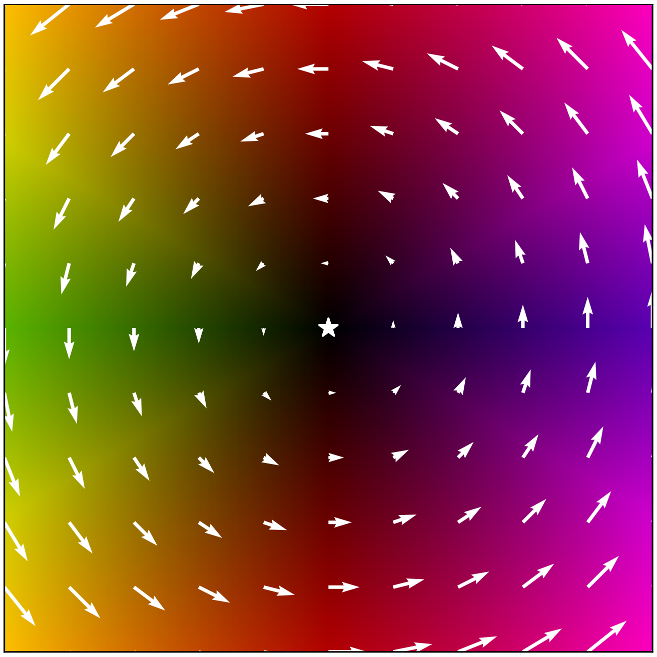

(b) Curl

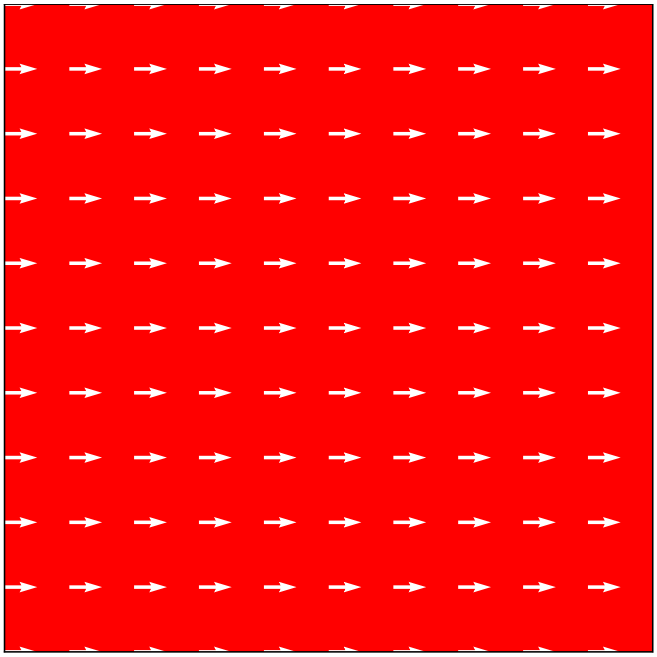

(c) Translation

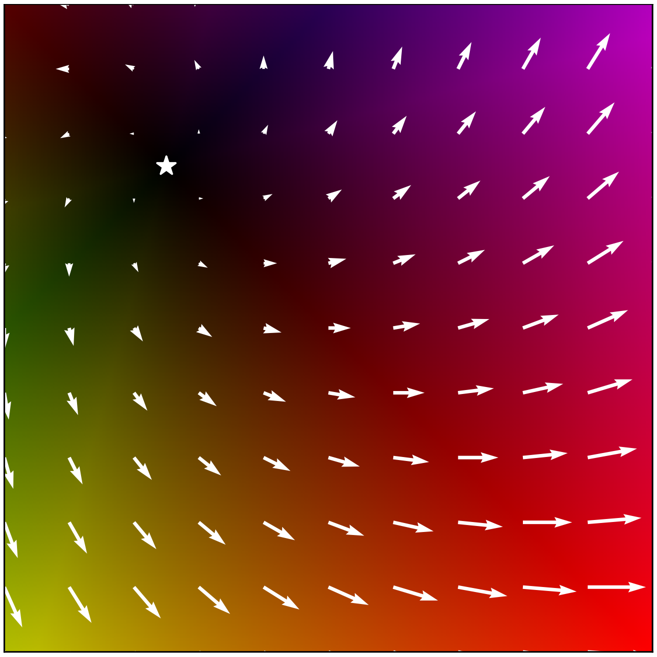

(d) Combined

Figure S1. Visualization of sparse and dense optic flow as a vector field. The figure shows theoretically constructed cases of optic flow using common visualization techniques. The continuous vector field can be color encoding, e.g. HSV color-encoding where the hue color represents the direction and saturation as the magnitude of the vector. The equivalent quiver plot for sparse sampling is overlaid on top of the dense visualization. The null points where optic flow points of zero vectors, more commonly referred to as the focus of expansion, are depicted as stars in the sparse optic flow field and black in the color-encoded dense optic flow field. The colorization of the dense optic flow is visually made symmetric around the vertical axis resulting in the same color red for vectors pointing leftward and rightward.

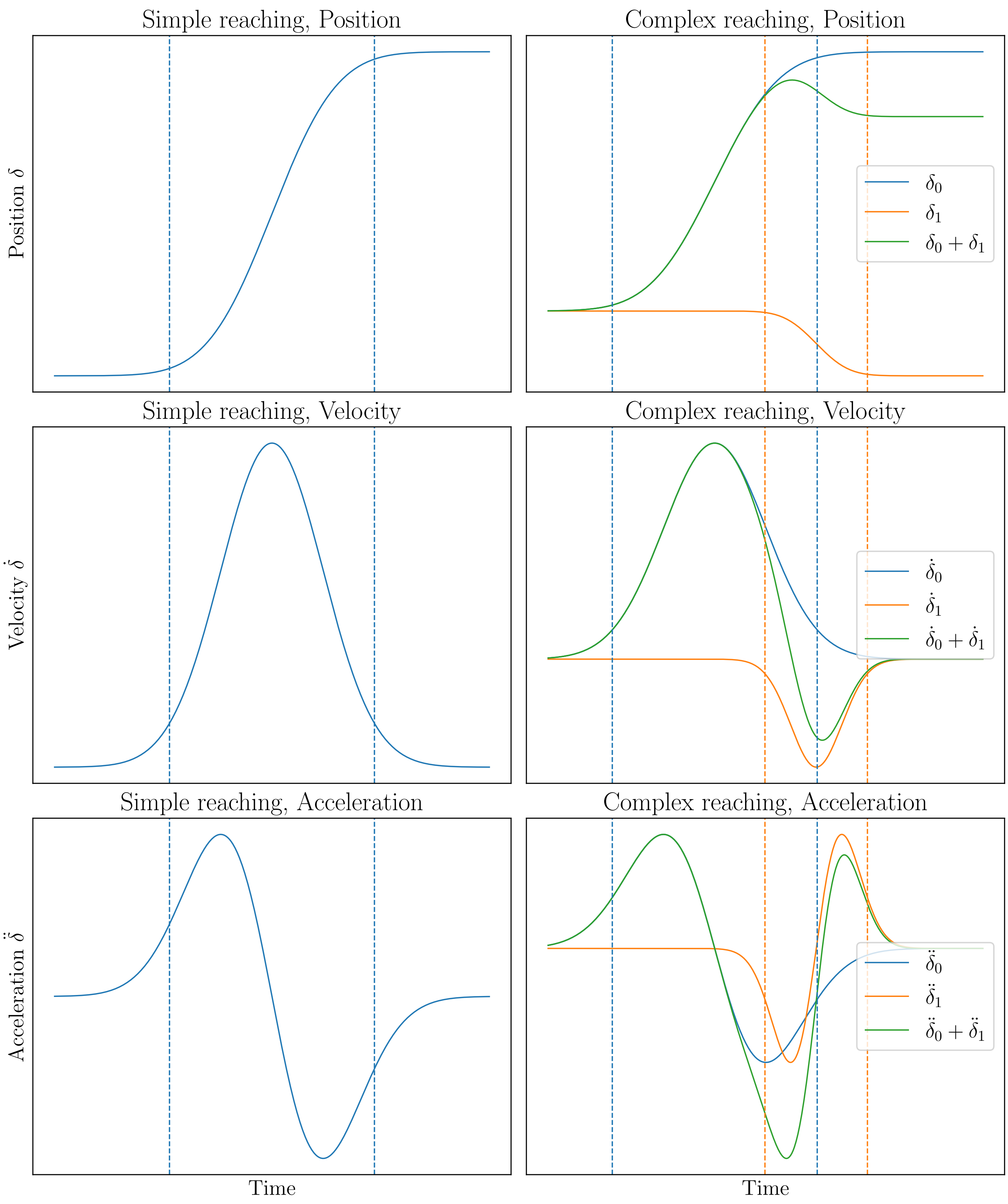

Figure S2. Theoretical intermittent control with ballistic corrections. The individual ballistic corrections are shown here as Gaussian functions with different correction strengths, modes of correction, and correction rates. These corrections are simple in isolation but can construct significant complex movements by overlapping the corrections through superposition as seen in the right column. The onset and offset timestamps are shown as vertical lines at 2.2 percent and 97.8 percent respectively of each correction movement distance, as defined in Eq. (8) .

Videos

Demonstration of an experimental trial

There are two videos provided demonstrating experimental trials conducted in this research. The videos are made for demonstration purposes only, showing one of the authors in one trial. The first video is composed of three panels: (a) The view shown to the participant, (b) the post-processed image with highlighted detected lane segment with the gaze point, and (c) the reconstructed retinal optic flow using color encoding (https://youtu.be/rW_X9OZ3mRM). The second video demonstrates a graphical user interface showcasing the post-processed data for the conducted experiment trial (https://youtu.be/d0xtk2d4wFk).

Tables

VR–HMD intrinsic calibration

In order to harmonize the image rendering in OpenVR and image processing in the Pupil software, an intrinsic calibration is needed to account for each optic lens in the HMD (one for each eye). These intrinsic calibration values are loaded in the firmware of the HMD device which may be accessed through the *SteamVR* API console, see Table S1 (only the images rendered for the left eye are considered). For implementation and a detailed explanation of how to apply these, refer to the tutorials offered by *OpenCV* (`https://docs.opencv.org/4.10.0/d4/d94/tutorial_camera_calibration.html`) and *Vive wave* (`https://hub.vive.com/storage/docs/en-us/DistortionCorrectionTheory.html`).

Table S1. Camera intrinsic calibration values for lenses inside the HMD. The intrinsic calibration values for a radial distortion camera model for our particular HMD used in the experiment. This is used to counteract the lens distortion phenomenon inside the head-mounted display.

Distortion coefficient	Left eye	Right Eye
k_1	-0.20518164004611725	-0.1938284587744417
k_2	-0.040357307230613204	-0.05225134475104416
p_1	0	0
p_2	0	0
k_3	-0.022284161177407984	-0.018314461950617165

Particle swarm optimization parameters

Table S2 presents parameters used in our particle swarm optimization implementation to identify the ballistic correction in the experimental steering wheel data.

Table S2. Particle swarm optimization meta-parameters and objective function parameter settings. The table presents the numerical parameter values used in the particle swarm optimization and the objective function for identifying the ballistic corrections.

Parameter	Value	Note
N_P	10000	Swarm population size
K	400	PSO max steps
α	1.0	Curve fitting parameter
γ	1.0	Time precision factor
Δt	1.0	Discrete time step size
v_max	1.1	Speed limit
w_max	1.4	Inertia (exploration)
w_min	0.3	Inertia (exploitation)
β	0.991	Inertia decay
c_1	3.0	Cognitive factor
c_2	$4.0 - c_1 = 1.0$	Social factor
K	400	Maximum number of steps in each PSO iteration
$N_{\delta,\mathrm{max}}$	6	Maximum Gaussian components for N_δ
S_min	200	Minimum fitness

Particle swarm optimization algorithm

Particle swarm optimization is one of many biological-inspired meta-heuristic optimization methods, designed to find local optima in a search space given an objective function. In contrast to classical optimization methods, these meta-heuristic optimization methods do not impose many formal strict requirements on the search space or objective (fitness) function such as continuity or differentiable. However it comes at the cost of inefficient computing, and the proposed solution is not guaranteed to be the global optimum. Since there are no real-time computing constraints and dealing with high-dimensional complex search space, the particle swarm optimization method is a suitable approach for our application.

Our implementation of particle swarm optimization is described in Alg. 1 where the sub-routines are described in detail in Alg. 2. Our implementation follows the standard implementation[1] with a few additional features of extending the search

space dimension by three for each iteration, due to each ballistic correction adding three new parameters. For convenience, the objective function is inverted so the optimization score returned by the function is maximized instead of minimized

$$\arg\max_{\boldsymbol{x}} g(\boldsymbol{x}) = \arg\min_{\boldsymbol{x}} f(\boldsymbol{x}), \text{ where } g(\boldsymbol{x}) = \frac{1}{f(\boldsymbol{x})}, \tag{S2}$$

where $f(\boldsymbol{x})$ is the objective to be minimized with respect to $\boldsymbol{x}$, resulting in maxizing $g(\boldsymbol{x})$. Specific numeric values used in this work for the meta-heuristic and objective function parameters can be found in Table S2.

Algorithm 1 Particle swarm optimization algorithm

Require: $S_{\min} > 0, N_{\max} > 0, \boldsymbol{x}_{\min} < \boldsymbol{x}_{\max} \in \mathbb{R}^3, g(\boldsymbol{x}) : \mathbb{R}^{3N} \to \mathbb{R}$ ▷ Minimum score, maximum iterations, and search space boundaries
Ensure: $\mathbb{R}^{3N} : \boldsymbol{x}^*, g(\boldsymbol{x}^*) > 0$ ▷ Candidate solution $\boldsymbol{x}^*$
procedure PARTICLESWARMOPTIMIZATION($z(\boldsymbol{t}), \boldsymbol{x}_{\min}, \boldsymbol{x}_{\max}$) ▷ Reference signal $z(\boldsymbol{t})$
  Converged ← False
  $N \leftarrow 1$
  while not Converged **do**
    $\mathbb{S} \leftarrow$ InitializeSwarm($N, \boldsymbol{x}_{\min}, \boldsymbol{x}_{\max}$)
    for $n \leftarrow 0, \dots, K-1$ **do**
      $\mathbb{S} \leftarrow$ EvaluateSwarm($\mathbb{S}, z(\boldsymbol{t})$)
      $\mathbb{S} \leftarrow$ UpdateVelocityPosition($\mathbb{S}$)
      $w \leftarrow \mathrm{Clamp}(\beta w, w_{min}, w_{max})$ ▷ Decay particle inertia, transform exploration→exploitation
    end for
    Criteria ← ($\mathbb{S}.p_{\text{best}}.s_{\text{best}} < p_{\text{candidate}}.s_{\text{score}}$) ▷ Candidate better than current swarm best
    Criteria ← Criteria **and** $p_{\text{candidate}}.s_{\text{score}} > S_{\min}$ ▷ Candidate fulfills minimum score
    Criteria ← Criteria **or** $N \geq N_{\max}$ ▷ Reached maximum computational iterations
    if Criteria **then**
      Converged ← True
    else
      $p_{\text{candidate}} \leftarrow \mathbb{S}.p_{\text{best}}$ ▷ Store the candidate solution
      $N \leftarrow N + 1$ ▷ Increase the dimensionality
    end if
  end while
  return $\boldsymbol{x}_{\text{candidate}} \leftarrow p_{\text{candidate}}.\boldsymbol{x}$
end procedure

Algorithm 2 Particle swarm optimization algorithm: functions

Require: $N > 0, \boldsymbol{x}_{\min} < \boldsymbol{x}_{\max}$ ▷ Dimensions, lower and upper bound for the search space
function INITIALIZESWARM($N, \boldsymbol{x}_{\min}, \boldsymbol{x}_{\max}$) ▷ Initialize the swarm of particles
  $\boldsymbol{x}_{\min} \leftarrow \text{repeat}(\boldsymbol{x}_{\min}, N)$ ▷ Repeat the array so the length is $N_{dim} \cdot N$
  $\boldsymbol{x}_{\max} \leftarrow \text{repeat}(\boldsymbol{x}_{\max}, N)$
  for $p_i \in \mathbb{S}$ **do** ▷ $\boldsymbol{r} = (r_0, \dots, r_{3N-1})$ randomly uniform sampled values $r_i \in [0,1]$
    $\boldsymbol{x}_i \leftarrow \boldsymbol{x}_{\min} + \boldsymbol{r} \odot (\boldsymbol{x}_{\max} - \boldsymbol{x}_{\min})$ ▷ Hadamard product $\odot$
    $\boldsymbol{v}_i \leftarrow \gamma \Delta t^{-1}(-\frac{\boldsymbol{x}_{\max} - \boldsymbol{x}_{\min}}{2} + \boldsymbol{r} \odot (\boldsymbol{x}_{\max} - \boldsymbol{x}_{\min}))$
    $p_i \leftarrow (\boldsymbol{x}_i, \boldsymbol{v}_i)$
  end for
  return $\mathbb{S}$
end function
Require: $\mathbb{S}, g(\boldsymbol{x}), z(\boldsymbol{t})$ ▷ Swarm, objective function, reference signal
function EVALUATESWARM($\mathbb{S}, z(\boldsymbol{t})$)
  for $p_i \in \mathbb{S}$ **do**
    $\boldsymbol{x} \leftarrow p_i.\boldsymbol{x}$
    $s \leftarrow g(z(\boldsymbol{t}), \boldsymbol{x})$ ▷ Objective score see Eq.(8)
    if $s > p_i.s_{\text{best}}$ **then** ▷ Store the personal best score
      $p_i.s_{\text{best}} \leftarrow s$
      $p_i.\boldsymbol{x}_{\text{best}} \leftarrow \boldsymbol{x}$
    end if
    if $s > \mathbb{S}.p_{\text{best}}.s_{\text{best}}$ **then** ▷ Store the swarm best score
      $\mathbb{S}_i.p_{\text{best}} \leftarrow p_i$
    end if
  end for
  return $S.p_{\text{best}}.s_{\text{best}}$
end function
Require: $\mathbb{S}, c_1, c_2, v_{\max}, w$ ▷ Particle swarm, cognitive factor, social factor, speed limit, current inertia
function UPDATEVELOCITYPOSITION($\mathbb{S}$)
  $\boldsymbol{x}_{\text{sb}} = \mathbb{S}.p_{\text{best}}.\boldsymbol{x}$ ▷ Swarm best
  for $p_i \in \mathbb{S}$ **do**
    $\boldsymbol{x} \leftarrow p_i.\boldsymbol{x}$
    $\boldsymbol{x}_{\text{pb}} \leftarrow p_i.\boldsymbol{x}_{\text{best}}$ ▷ Personal best
    $\boldsymbol{v} \leftarrow p_i.\boldsymbol{v}$
    $\boldsymbol{v} \leftarrow w\boldsymbol{v} + c_1 r_1 \frac{\boldsymbol{x}_{pb} - \boldsymbol{x}}{\Delta t} + c_2 r_2 \frac{\boldsymbol{x}_{sb} - \boldsymbol{x}}{\Delta t}$ ▷ Update velocity with uniform random sample $r_i \in [0,1]$
    if $||\boldsymbol{v}||_2 > v_{\max}$ **then** ▷ Restrict velocity threshold value
      $\boldsymbol{v} \leftarrow v_{\max} ||\boldsymbol{v}||_2^{-1} \boldsymbol{v}$
    end if
    $\boldsymbol{x} \leftarrow \boldsymbol{x} + \boldsymbol{v}\Delta t$
    $p_i.\boldsymbol{x} \leftarrow \boldsymbol{x}$
    $p_i.\boldsymbol{v} \leftarrow \boldsymbol{v}$
  end for
  return $\mathbb{S}$
end function

Approximating asymmetric reaching movement with trailing heavy tail as symmetric

As detailed in the main article, experimental observation reveals that human reaching is often asymmetric with a trailing heavy tail. This describes the reaching movement as slower homing when approaching the destination or the reaching target. Plamandon presents a theoretical model in their work[2] where a single heavy tail ballistic correction can be described using a log-normal characteristic bell-curve

$$\dot{\hat{\delta}}_{\mathrm{b}}(P(t,t_{\mathrm{on}},t_{\mathrm{off}}),a_{\mathrm{I}},\mu_{\mathrm{I}},\sigma_{\mathrm{I}}) \triangleq \frac{a_{\mathrm{I}}}{P(t)} \exp \frac{-(\ln(P(t))-\mu_{\mathrm{I}})^2}{2\sigma_{\mathrm{I}}^2}, \tag{S3}$$

where

$$P(t,t_{\mathrm{on}},t_{\mathrm{off}}) = \frac{t-t_{\mathrm{on}}}{t_{\mathrm{off}}-t_{\mathrm{on}}}, \quad \text{for} \quad t_{\mathrm{on}} \leq t \leq t_{\mathrm{off}} \tag{S4}$$

$t_{\{\mathrm{on,off}\}}$ are some arbitrary onset and offset timings for the reaching correction defined by Plamandon (different from our definitions). Despite better approximation according to their work, the application of log-normal convolutes the direct interpretation of the theoretical model. The resulting strength and the mode in the correction can no longer be easily deduced in the presented model. Furthermore, the model requires additional onset and offset timing parameters to describe the heavy tail model, totaling five parameters per reaching correction in contrast to three for symmetric.

The discrepancies between a symmetric and asymmetric heavy-tailed reaching correction are illustrated in Fig. S3b using PSO to minimize the error of the two. The heavy-tail property mainly shifts the symmetric mode towards the tail and increases the spread (variance or correction speed parameter) of the Gaussian function. In this work, the correction onset is a central component of the analysis and is directly affected by the mode and correction speed parameter and further concern may arise. However, in this approximation, the shift of mode and increase of correction speed parameters partly cancel each other out, limiting approximation error for the onset. However, the same can not be argued about the offset timing. The strength of the correction is largely unaffected by the approximation.

Interestingly, as seen in Fig. S3c, the heavy-tail feature can be captured by multiple smaller overlapping symmetric Gaussian functions. It raises the question of whether the heavy-tail property observed in the experimental data in early literature, as described by Plamandon[2], may in reality be a train of smaller overlapping corrections instead. This investigation is however outside the scope of this work.

Idealization of retinal optic flow on the assumption of exact smooth pursuit

This work heavily rests on the idealization of retinal optic flow as perceived by the research participant by assuming "exact" smooth pursuits. The idealization allows the efficient modeling and computational estimation of retinal optic flow. This assumption of exact smooth pursuit implies that the optic flow at the fovea in the retina, corresponding to the gaze point in the image space, is nulled. This is explicitly exploited in Eq. (14) where retinal optic flow is computed using estimated head-fixed optic flow and gaze data. Our numerical retinal optic flow estimation method is comparable to the works of Matthis and colleagues[3] and uses similar, if not the same, assumptions of exact smooth pursuits.

The smooth pursuits were yielded from *naive segmented linear regression* (NSLR)[4], which are then compared to the yaw angular velocity of the vehicle. The refactored NSLR implementation C++ source code is available at `https://gitlab.com/bjornborg/nslr`. The head rotation velocity of the research participants is neglected from the computation since it is relatively small compared to that of the vehicle. As predicted in the theories of Kim and Turvey[5], Wann and colleagues[6], and further experimentally shown by Lappi and colleagues[7], the exact smooth pursuits should show that the horizontal gaze velocity is a half of the yaw angular velocity of the vehicle, i.e. a slope of slope $= -2.0$. The mathematical proof is provided in the next section, where the gaze angle is mirrored. The experimental data from our work show good correspondence with this, see Fig. S4. Our linear regression slope was estimated to be slope ≈ -2.36, which is comparable to (slope ≈ -2.53) found in the work of Lappi and colleagues[7]. Since naive linear regression is sensitive to data outliers, a simpler outlier rejection method using *random sampling consensus*[8] (RANSAC) has been applied. Thus an experimental smooth pursuit gain 0.8475 is observed in our experimental data.

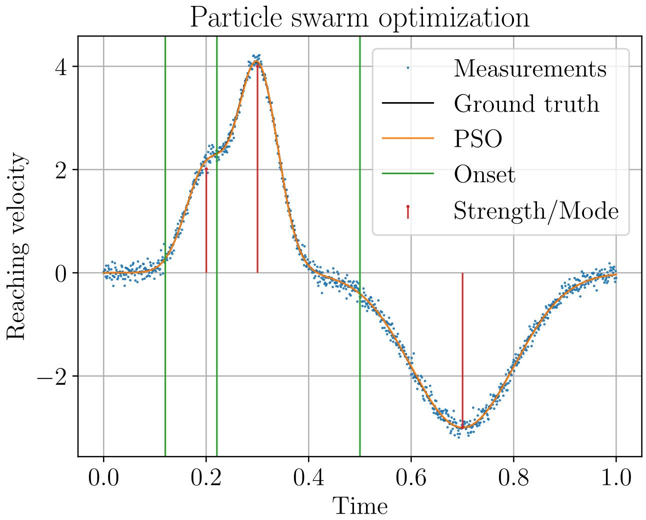

(a) Symmetric bell-shaped correction

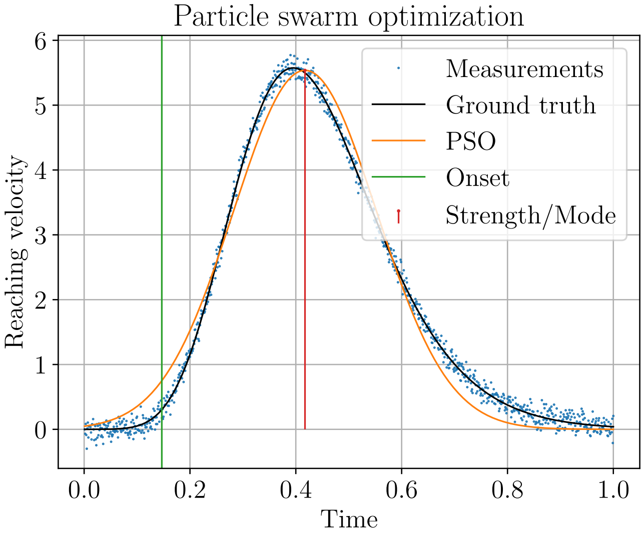

(b) Heavy-tailed bell-shaped correction with one Gaussian

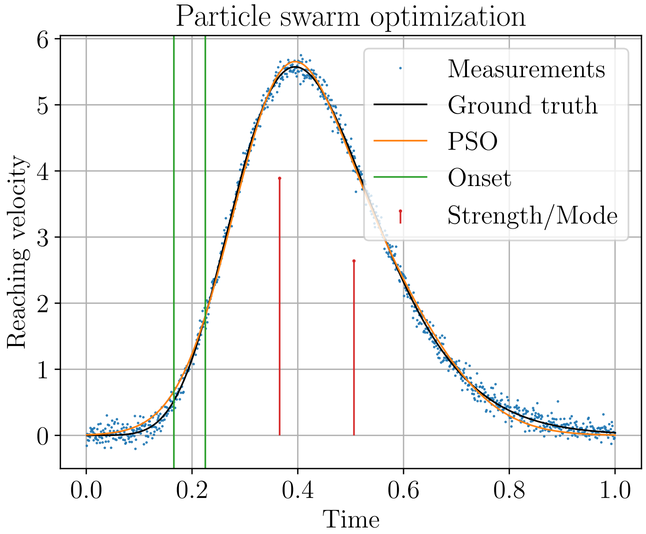

(c) Heavy-tailed bell-shaped correction with two Gaussian

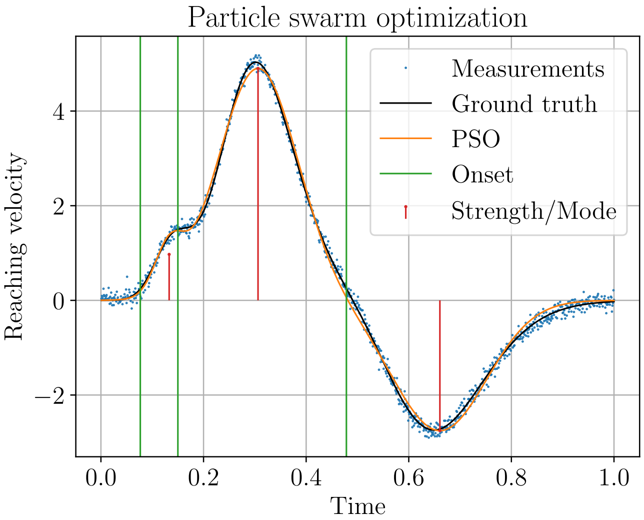

(d) Heavy-tailed bell-shaped corrections

Figure S3. Identifying reaching correction using PSO in synthetic data. The figure shows the proof of concept of identifying reaching corrections using PSO and the performance in various scenarios. Panel **(a)** shows how well PSO performs when applied to synthetic data. Here the data is constructed with three overlapping symmetric Gaussian functions, $a_{\mathrm{I},\{0,1,2\}} \in \{2.0, 4.0, -3.0\}$, $\mu_{\mathrm{I},\{0,1,2\}} \in \{0.20, 0.30, 0.7\}$, and $\sigma^2_{\mathrm{I},\{0,1,2\}} \in \{0.00159572, 0.00159706, 0.01\}$. Panel **(b)** shows the discrepancies when approximating a heavy-tail bell-shaped curve $a_\mathrm{I} = 2.0$, $\mu_\mathrm{I} = -1.0$, $\sigma^2_\mathrm{I} = 0.05$, $t_\mathrm{on} = -0.2$, and $t_\mathrm{off} = 1.5$ as defined in Eq. (S3), to a single symmetric Gaussian function using PSO. Panel **(c)** shows how a heavy-tail bell-shaped curve, the same as in panel **(b)**, may be approximated by two smaller overlapping Gaussian functions capturing the heavy tail feature. Panel **(d)** shows the similar (not equivalent) complex movement as (a) but heavy-tailed reaching corrections $a_{\mathrm{I},\{0,1,2\}} \in \{0.8, 1.5, -1\}$, $\mu_{\mathrm{I},\{0,1,2\}} \in \{-0.5, -1.2, -1.0\}$, and $\sigma^2_{\mathrm{I},\{0,1,2\}} \in \{0.005, 0.02, 0.02\}$, $t_{\mathrm{on},\{0,1,2\}} = \{-0.4, -0.2, 0.0\}$, and $t_{\mathrm{off},\{0,1,2\}} = \{0.5, 1.5, 1.8\}$. All synthetic data are padded with a normal distribution measurement noise $n(t) \sim \mathcal{N}(0, 0.1)$. For the application of real experimental steering wheel data, refer to Fig. (7) .

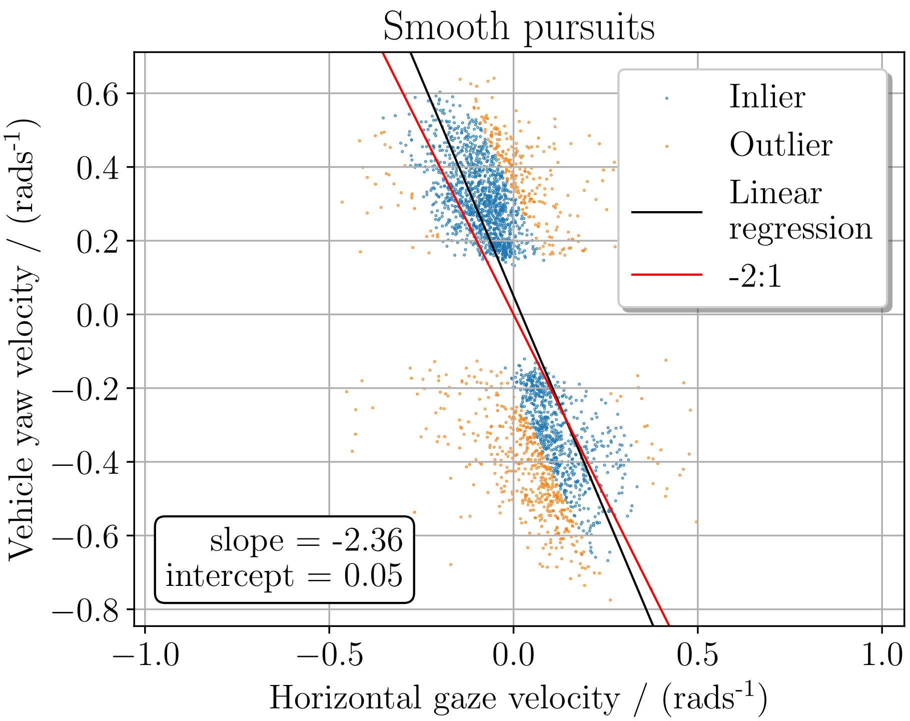

Figure S4. Relating horizontal gaze velocity to vehicle yaw velocity. The gazing behavior of research participants in the curve is shown in the figure. The horizontal gaze velocities and the corresponding half of the vehicular yaw angular velocity are sampled for a smooth pursuit. A naive linear regression is applied to the experimental inlier data yielding a slope of -2.36, compared to the theorized gaze fixation predicting -2.0. An outlier rejection using RANSAC has been applied before computing the linear regression.

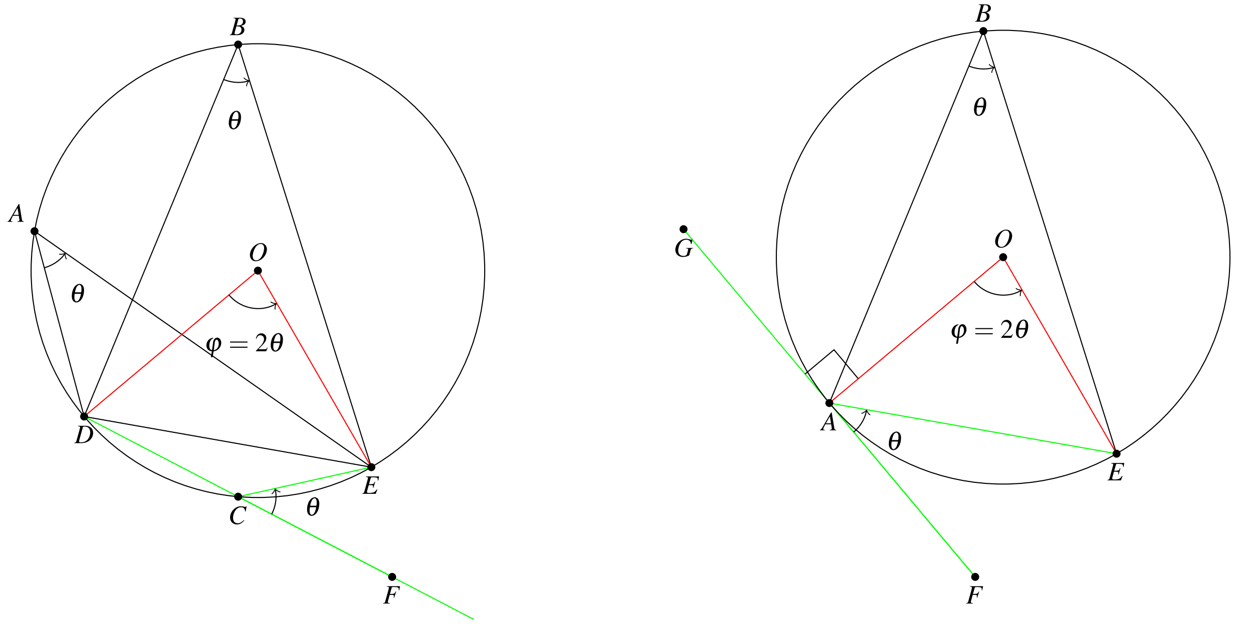

(a) Inscribed angle theorem

(b) Special case of a tangent

Figure S5. Geometrical proof relating vehicular yaw angular and horizontal gaze velocities. The figure shows the geometrical proof relating all the angles when gazing at a point on the curvilinear path.

Proof relating horizontal gaze and vehicular yaw angular velocity

Using the *inscribed angle theorem*, as shown in Fig. S5a, it can be stated that

$$\varphi = 2\theta \tag{S5}$$

for any point A in the set of points M which the angle $\angle DAE = \theta$ can be constructed on the circular arc. The complement set M^c, which is the complement arc to complete the circle, and the supplementary angle $\angle DCE = \theta$ can be constructed as illustrated in Fig. S5a at point C.

Let the points A and C converge to the point D from each side which establishes the special case of the inscribed angle theorem of a tangent AF where $\angle FAE = \angle FDE = \angle FCE = \theta$, as shown in Fig. S5a. This is the approximate case (disregarding the height dimension) when the agent fixates their gaze at point E on the circular path from their point A which establishes the horizontal gaze angle θ while the heading of the vehicle is the tangent AF. The time derivative of Eq. (S5) is thus simply

$$\dot{\varphi}(t) = 2\dot{\theta}(t) \tag{S6}$$

where $\dot{\varphi}(t)$ is the vehicular yaw angular velocity and $\dot{\theta}(t)$ is the horizontal gaze velocity.

References

1. Wahde, M. *Biologically inspired optimization methods: an introduction* (WIT press, 2008).
2. Plamondon, R. On the origin of asymmetric bell-shaped velocity profiles in rapid-aimed movements. In Requin, J. & Stelmach, G. E. (eds.) *Tutorials in Motor Neuroscience*, 283–295, DOI: https://doi.org/10.1007/978-94-011-3626-6_23 (Springer Netherlands, Dordrecht, 1991).
3. Matthis, J. S., Muller, K. S., Bonnen, K. L. & Hayhoe, M. M. Retinal optic flow during natural locomotion. *PLOS Comput. Biol.* **18**, 1–37, DOI: https://doi.org/10.1371/journal.pcbi.1009575 (2022).
4. Pekkanen, J. & Lappi, O. A new and general approach to signal denoising and eye movement classification based on segmented linear regression. *Sci. reports* **7**, 17726, DOI: https://doi.org/10.1038/s41598-017-17983-x (2017).
5. Kim, N.-G. & Turvey, M. Eye movements and a rule for perceiving direction of heading. *Ecol. Psychol.* **11**, 233–248, DOI: https://doi.org/10.1207/s15326969eco1103_3 (1999).
6. Wann, J. P. & Swapp, D. K. Why you should look where you are going. *Nat. neuroscience* **3**, 647–648, DOI: https://doi.org/10.1038/76602 (2000).
7. Lappi, O. *et al.* Humans use optokinetic eye movements to track waypoints for steering. *Sci. reports* **10**, 4175, DOI: https://doi.org/10.1038/s41598-020-60531-3 (2020).
8. Fischler, M. A. & C, B. R. Random sample consensus: a paradigm for model fitting with applications to image analysis and automated cartography. *Commun. ACM* **24**, 381–395, DOI: https://doi.org/10.1145/358669.358692 (1981).
